# Repurposing live attenuated trivalent MMR vaccine as cost-effective cancer immunotherapy

**DOI:** 10.3389/fonc.2022.1042250

**Published:** 2022-11-09

**Authors:** Yuguo Zhang, Musa Gabere, Mika A. Taylor, Camila C. Simoes, Chelsae Dumbauld, Oumar Barro, Mulu Z. Tesfay, Alicia L. Graham, Khandoker Usran Ferdous, Alena V. Savenka, Jean Christopher Chamcheu, Charity L. Washam, Duah Alkam, Allen Gies, Stephanie D. Byrum, Matteo Conti, Steven R. Post, Thomas Kelly, Mitesh J. Borad, Martin J. Cannon, Alexei Basnakian, Bolni M. Nagalo

**Affiliations:** ^1^ Department of Pathology, University of Arkansas for Medical Sciences (UAMS), Little Rock, AR, United States; ^2^ Department of Molecular Medicine, Mayo Clinic, Rochester, MN, United States; ^3^ The Winthrop P. Rockefeller Cancer Institute, University of Arkansas for Medical Sciences (UAMS), Little Rock, AR, United States; ^4^ Department of Pharmacology and Toxicology, University of Arkansas for Medical Sciences (UAMS), Little Rock, AR, United States; ^5^ School of Basic Pharmaceutical and Toxicological Science, College of Pharmacy, University of Louisiana at Monroe, Monroe, LA, United States; ^6^ Public Health Department, AUSL Imola, Imola, Italy; ^7^ Department of Microbiology and Immunology, University of Arkansas for Medical Sciences (UAMS), Little Rock, AR, United States

**Keywords:** live attenuated vaccine, hepatocellular carcinoma, colorectal cancer, measles, mumps, rubella (MMR) vaccine, immunotherapy, oncolytic viral therapy

## Abstract

It has long been known that oncolytic viruses wield their therapeutic capability by priming an inflammatory state within the tumor and activating the tumor immune microenvironment, resulting in a multifaceted antitumor immune response. Vaccine-derived viruses, such as measles and mumps, have demonstrated promising potential for treating human cancer in animal models and clinical trials. However, the extensive cost of manufacturing current oncolytic viral products makes them far out of reach for most patients. Here by analyzing the impact of intratumoral (IT) administrations of the trivalent live attenuated measles, mumps, and rubella viruses (MMR) vaccine, we unveil the cellular and molecular basis of MMR-induced anti-cancer activity. Strikingly, we found that IT delivery of low doses of MMR correlates with tumor control and improved survival in murine hepatocellular cancer and colorectal cancer models *via* increased tumor infiltration of CD8+ granzyme B+ T-cells and decreased macrophages. Moreover, our data indicate that MMR activates key cellular effectors of the host’s innate and adaptive antitumor immunity, culminating in an immunologically coordinated cancer cell death. These findings warrant further work on the potential for MMR to be repurposed as safe and cost-effective cancer immunotherapy to impact cancer patients globally.

## Introduction

In the past few years, immunotherapy has gained momentum in cancer treatment by enabling improvements in the survival of patients with advanced cancers (1–6). Oncolytic viral therapy is an emerging new class of cancer immunotherapy that involves selectively infecting and killing tumor cells (7–10). Oncolytic viruses (OVs) induce versatile and multimodal antitumor activity involving innate and adaptive immunity (11, 12). This unique ability, shared among viruses and bacteria, has been exploited to develop vaccines and anticancer agents (13). Several OVs have already exhibited promising potential for treating human cancers (14–17). Despite the evidence of therapeutic benefits (18, 19), seamless clinical use of OVs in cancer patients also faces numerous challenges. These limitations comprise the varying efficacy of OVs, mostly attenuated wild-type or genetically modified single viral vectors, in human cancers (4). More importantly, the high cost of developing and manufacturing current OVs makes them far out of reach for patients with lower socioeconomic status (20). For these reasons, developing economically sustainable and effective analogous approaches to current high-cost OVs is an area of high interest in oncology.

Recent studies have suggested that one exciting approach to expand the clinical benefit of OVs to many patients is to repurpose live attenuated viral vaccines (LAVs) for cancer immunotherapy (21–23). However, most of these studies have not focused on LAVs as monotherapies but highlighted their role as adjuvant therapies in animal studies (21, 22, 24–29). In addition, these strategies require a capital-intensive production pipeline, and thus a considerable limitation to making this approach available to all patients amenable to anti-cancer immunotherapy.

The trivalent live attenuated measles, mumps, and rubella viruses (MMR) vaccine is attractive as an immunovirotherapy primarily for its proven safety record but has other useful features as well. MMR has been shown to stimulate a potent and long-lasting protective immunity against measles, mumps, and rubella in humans (30–35). Moreover, studies have shown that vaccine lineages of measles and mumps viruses exert effective cytotoxicity against human tumors in cell cultures, animal models, and clinical trials (24, 26, 27, 36, 37). Furthermore, MMR is readily available, low-cost, and accessible worldwide. Its use requires no regulatory approval from the US Food and Drug Administration (FDA).

To gain insight into the antitumor mechanism of MMR, we studied the impact of intratumoral administration of multiple low doses of MMR in animal models of hepatocellular carcinoma (HCC) (38–41) and colorectal cancer (CRC). We selected these two solid tumor models because the liver is one of the dominant metastatic sites for colorectal cancer cells (1, 42, 43). In addition HCC and CRC (44, 45) are among the cancers with highest unmet clinical need in oncology. Measles, mumps, and rubella have been shown to infect but not extensively replicate in mouse cancer cells (46–49). And OVs promote antitumor immunity against infected and uninfected cancer cells (50–54). Thus, we employed an integrated transcriptomic and proteomic approach to elucidate the changes in the proteome and transcriptome of murine tumors in response to MMR-based IT immunotherapy. These findings demonstrate that low doses of MMR modulate an immune response that culminates in significant tumor growth delay and extended survival in animal models, warranting its further evaluation as potential cost-effective cancer immunotherapy.

## Results

### Low MMR doses induce modest cytotoxicity in murine and human HCC cell lines

**Figure 1:**
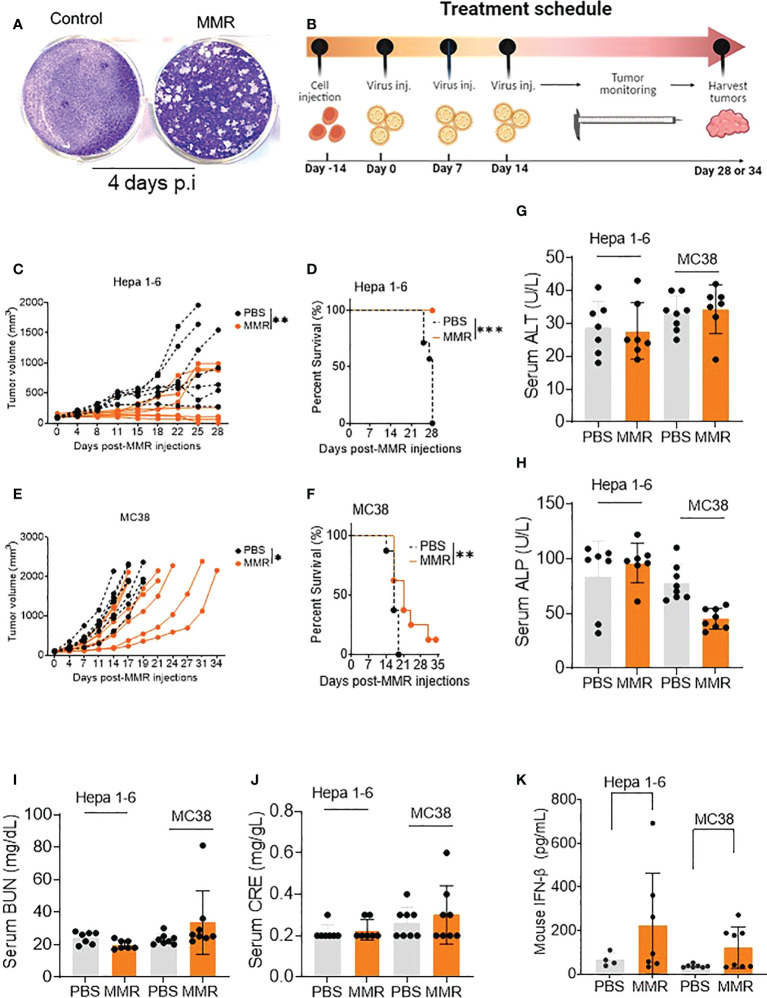
IT injections of MMR induce tumor growth delay and improve survival in murine tumor models. **(A)** A single dose of MMR (1x 102 TCID50) was used to infect a monolayer of Vero cells (2.5 x 105/well in 6-well plates) four days later, cells were stained with crystal violet to reveal infectious viral plaques. **(B)** In the treatment schedule, at day -14, tumor cells were subcutaneously implanted into the right flanks of mice, then PBS or MMR was injected into tumor-bearing mice at days 0, 7, and 14. Tumors were harvested at different time points throughout the study. **(C–F)** Three doses (days 0,7 and 14) of PBS or MMR were intratumorally (IT) administered into mice bearing subcutaneous Hepa 1-6 and MC38 tumors. Individual tumor growth curves are shown (n =7/group [Hepa 1-6] n=8/group [MC38]) **(C, E)**. Paired t test with and Wilcoxen signedrank test were performed (95% CI), ***P<0.0005, **P<0.005, *P<0.05. The day when we injected the first MMR or PBS into the mice is defined as day 0. Kaplan–Meier survival analysis of subcutaneous Hepa 1-6 and MC38 tumor-bearing mice **(D, F)**. The statistical significance of differences in the survival curves between the groups was evaluated using the log-rank (Mantel-Cox) test. **(G–J)** Mouse Serum biochemical analysis. Serum chemistry analysis of markers of liver and nephrotoxicity. Graphs showing changes in concentration of enzymes such as alanine aminotransferase (ALT, **G**), alkaline phosphates (ALP, **H**), blood urea nitrogen (BUN, **I**), serum creatinine (CRE, **J**) between the PBS and MMR-treated groups. **(K)** Levels of mouse type I interferon b (IFN- b) in serum. Level of antiviral cytokine (IFN- b) was measured in serum from mice treated with MMR or PBS.

To determine whether a low dose of MMR contains infectious measles, or mumps, or rubella particles, we first infected a monolayer of Vero cells with a single dose of 1 x 10^2^ TCID_50_ (for each virus) of MMR in a 6-well plate for four days. After the incubation period, we stained infected cells with crystal violet revealing that infection with at least one or all three viruses induced oncolysis (**Figure 1A**). In addition, a cell viability assay was used to investigate whether low doses of MMR can induce oncolysis in murine and human HCC cell lines. Unsurprisingly, an infectious titer of 1000 TCID_50_ (infants’ dose of MMR vaccine 15 months to 12 years old) of MMR produced less than 20% cancer cell death in both Hepa 1-6 and Hep3B cells (**Supplementary Figures 1A, B**). These results show that at least one or all the viruses in the MMR formulation can effectively infect murine and human HCC cells but do not cause extensive cytopathic effects.

### IT injections of a low dose of MMR induce subcutaneous tumor growth delay and improve survival in mouse tumor models

Administration of low doses of attenuated oncolytic viruses has been widely used to reduce the risk of severe toxicity, environmental shedding, or reversion to the wild-type phenotype (55). Therefore, we performed three IT injections (days 0, 7, and 14) of 50 µL of phosphate-buffered saline (PBS; PBS control groups) or low doses of MMR (1 × 10^2^ TCID_50_ for each virus; MMR groups) into two subcutaneous mouse models of HCC (Hepa 1-6) and CRC (MC38) (**Figure 1B**). Following the above treatment schedule, we found that multiple injections of 1 × 10^2^ TCID_50_ of MMR resulted in significant tumor growth delay and extended survival compared to that in the control group (PBS) in Hepa 1-6 (tumor, *p=0.003*; survival, *p<*0.001; **Figures 1C, D**) and MC38 (tumor, *p=0.02*; survival, *p=0.01*; **Figures 1E, F**) models. In comparison, the IT dose (1 × 10^2^ TCID_50_) of MMR in this study is 10^4^- 10^5^-fold lower than that of oncolytic measles and mumps (i.e., 10^7^-10^8^ TCID_50_) currently used in mouse tumor models and clinical trials (24, 26, 27, 36, 37). Studies have shown that the intracranial administration of the measles vaccine strain can lead to severe adverse events, often lethal in transgenic mice constitutively expressing the measles vaccine receptor human vaccine receptor (SLAM) (56). Moreover, fatal measles infections have been reported in immunocompromised vaccinated children (57) and adults with acquired immunodeficiency syndrome (58). However, following three IT injections, no change in body weight (>20% weight loss) or severe adverse events was observed in the MMR-group (**Supplementary Figures 1C, D**). However, in the PBS-group, two mice reached tumor burden (>2,000 mm (3)) on days 22 and 25, and the remaining mice showed ulcerations in their tumors by day 28. In both the Hepa 1-6 and MC-38 cohorts, serum was collected from several mice at different time points to perform serum chemistry analysis to determine if multiple doses of MMR induce severe toxicity, including hepatotoxicity and kidney toxicity. As expected, there was no significant changes between PBS and MMR in markers for liver toxicity (i.e., alanine aminotransferase [ALT], alkaline phosphatase [ALP]) or nephrotoxicity (i.e., creatinine [CRE], blood urea nitrogen [BUN]) in the two groups (PBS and MMR) (**Figures 1G–J** and **Supplementary Figures 2A–K**). Normal values of mouse blood biochemical markers are listed in **Supplementary Figures 2L, M**. There were increased levels of interferon-beta (IFN-β) in the serum of mice treated with MMR than PBS, suggesting activation of antiviral immune mechanisms (**Figure 1K**) (59). These findings indicate that multiple IT low doses of MMR are effective against murine HCC and CRC and without the side effect observed with other oncolytic viruses (i.e., hepatotoxicity and nephrotoxicity) (55, 60).

### Evaluation of tumor cell death induced by IT administrations of MMR

We performed a TUNEL assay to evaluate the difference in cell death induced by MMR IT injections versus vehicle (PBS) controls in Hepa 1-6 tumors. Tumors were harvested in both cases at the end of the experiment (day 28) when the acute phase of cell death induced by MMR had likely already passed. This was done because we aimed at tumor size and animal survival measurements, which were our primary endpoint. Several reasons could cause cell death measured by TUNEL assay. First, it is a natural cell turnover, which can be expected in both treated and untreated tumors and normal tissues. Second, mechanical damage from the injection needles – in both groups. Third is hypoxia-induced tumor necrosis. This usually occurs due to insufficient vascularization, especially in large tumors, and is closer to the tumor’s center. These TUNEL-positive cells were expected to be more abundant in PBS-treated tumors, primarily because they were harvested at late stages from moribund animals. The fourth potential cause is the MMR injection expected to induce an immune response. The overlap of these three causes was expected not to create a significant difference between vehicle and MMR-treated samples. We found that the overall percentage of TUNEL-positive cells was high in both groups, at ~5-10% of total cells. As expected, our experiment showed that TUNEL-positivity measured in mean intensity of color in MMR-treated tumors was only slightly higher than in vehicle-treated samples (**Supplementary Figure 3A**). Representative images are shown in **Supplementary Figures 3B, C**. The deviations within areas in the same tumor and between the tumors were also very high. As a result, there was no statistical difference between the two groups. The TUNEL pattern was mainly nuclear indicative of apoptosis rather than necrosis, although “classic” apoptotic nuclear fragmentation was rarely observed.

### Measles, mumps, and rubella viruses likely infect but do not extensively replicate in murine tumors

To assess whether MMR-induced antitumor activity is due to viral replication and oncolysis, we amplified viral genes using specific primers to genes coding for measles nucleoprotein, mumps matrix protein, rubella envelope protein and murine β-actin in Hepa 1-6 and MC38 tumors. We did not detect viral genes in Hepa 1-6 and MC38 tumors, but murine β-actin was successfully amplified (**Supplementary Figure 3D**). These results show that the antitumor activity induced by MMR may be independent of direct and extensive viral oncolysis and replication, as demonstrated for other oncolytic viruses (61).

### IT administration of MMR increased the frequency of tumor-infiltration immune cells

**Figure 2:**
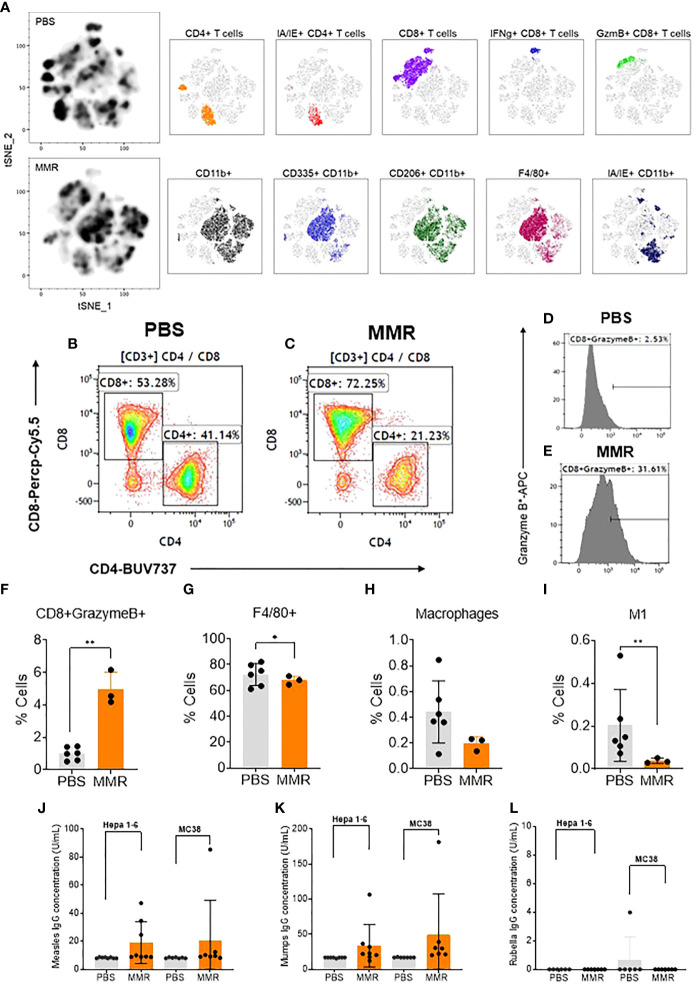
Analysis of tumor-infiltrating immune cells following intratumoral injection of MMR vaccine. **(A)** T-distributed stochastic neighbor embedding (tSNE) analysis showed a relatively dense intratumoral population of murine immune cells in Hepa 1-6 tumors treated with MMR vaccine compared to controls (PBS). We performed the tSNE plot on 10,000 downsampled events from viable CD45+ population per sample. The submyeloid population representing macrophages are CD11b+F480+ cells, and the two markers used to segregate the M1 and M2 sub-populations are CD206 and I-A/I-E, known as major histocompatibility class II (MHCII) in mouse. An example of gating shows two subpopulations based on their CD8+ and CD4+ T-cells frequencies between tumors treated with PBS **(B)** or MMR **(C)**. Histograms illustrating the increased frequency of tumor-infiltrating CD8+ granzyme B+ reactive CTLs in MMR compared to PBS **(D, E)**. Graphs showing the significant increase in CD8+ granzyme B+ **(F)** and reduction of F4/80+ macrophages **(G)**, total macrophages **(H)**, and inflammatory M1 macrophages **(I)** in the MMR group compared to PBS. The Bartlett test was used to test homogeneity of variance and normality. If the p value of the Bartlett test was no less than p=0.05, ANOVA and a two-sample t test were used to compare group means. If the p value of the Bartlett test was less than 0.05, the KruskalWallis and Wilcoxon rank-sum tests were used to compare group means. The figures demonstrate the potential significant difference of the gated subsets in the CD45+ population, determined by the p value. Quantification of virus-specific Immunoglobulin in serum. Blood was processed to serum to measure the levels of specific immunoglobulin (IgG) to measles **(J)** (negative < 8 U/mL positive > 10 U/mL), mumps measles **(K)** (negative < 17 IU/mL positive > 20 IU/mL), and rubella measles **(L)** (negative < 10 IU/mL equivocal 10-15 IU/mL positive > 15 IU/mL) in the MMR vs. PBS treated group. *P=0.03, **P=0.02.

To determine the various aspects of the immune response associated with IT administrations of MMR *in vivo*, Hepa 1-6 tumors were surgically removed and dissociated into single-cell suspension (see *Materials and methods*) (62). A multicolor flow cytometry assay was used to identify and assess the frequency of tumor-infiltrating (TILs) immune cells (e.g., CD3 [T cells], CD8 [T cells], CD4 [helper T cells], CD11b [dendritic cells], CD206 and I-A/I-E [F4/80 macrophages], CD335 [NK cells]). In comparison to IT injection of PBS, IT injection of MMR was associated with increased tumor infiltration of immune cells, particularly cytotoxic (CD8+) T cells and CD11b cells comprising active and memory virus specific CTLs (**Figures 2A–C**) (63, 64). Among the subset of CD8+ T cells the frequency of the CD8+ GranzymeB+ TILs population (p=0.02) was significantly upregulated in tumors treated with MMR compared to PBS (**Figures 2D–F**). There was no difference in the frequency of CD4+ (CD44+, IFNg+, Ki67+, PD-1+) (Supplementary Fig 4C–G). By contrast mice that received IT injections of MMR had decreased numbers of F4/80 macrophages (**Figure 2G**; *p=0.03*), total macrophages (**Figure 2H**; *p=0.05, not significant*) and M1 macrophages (**Figure 2,** M1 *p*=0.02). M1 macrophages have been associated with resistance to pathogens and inhibition of carcinogenesis, but the M2 phenotype exerts immunosuppressive effects (65, 66). Although not statistically significant, there was a decrease in the frequency of total macrophages, including M2 phenotype and NK cells in the MMR group compared to PBS controls (**Supplementary Figures 4I, M**). Interferon-gamma (IFNg+) is a potent antiviral cytokine that is essential for cytotoxic T cell-mediated elimination of measles, mumps, and rubella viruses and the establishment of antiviral immunity (67–69). There was no difference in the subsets of CD8+ IFNg+ and CD4+ IFNg+ between the MMR-treated and PBS-treated mice, indicating an absence of extensive viral replication or clearance of viral particles. CD8+ and CD4+ T lymphocytes are essential effectors of adaptive antiviral immune response (70). Previous studies showed that depletion of CD8+ T cells in primates exposed to wild-type measles is associated with severe disease (extensive rash, higher viral loads, and persistent viremia) (71, 72). Moreover, it has long been reported that children with a defect in T-lymphocyte activity often develop fatal diseases (73). Measles, mumps, and rubella vaccinations elicit potent humoral and cellular immune responses (68, 74–76). In the absence of viral replication (46–49), these results suggest that IT administration of low doses of MMR can modulate the immune compartment of the tumor microenvironment by increasing the subset of CD8+ GranzymeB+ TILs and decreasing the frequency of macrophage populations within the tumor.

### Evaluation of measles, mumps, and rubella specific antibodies after IT injections of MMR

To evaluate the adaptive immune response to measles, mumps, and rubella viruses, we measured the level of virus-specific immunoglobulin gamma (IgG) in serum following IT immunotherapy with MMR in the Hepa -16 and MC38 models. Interestingly, we found robust antibody responses to measles mumps viruses with high levels of IgGs, but not to rubella virus in MMR treated mice as compared to PBS (Figs. 2j, k, l). Human vaccine studies have reported a broad spectrum of differences in serum antibodies levels to rubella among vaccinated individuals, including waning or low antibodies responses (77). Moreover, reports have shown that antibodies targeting measles hemaglutinin (H) protein can increase its cellular uptake rather than inhibit viral entry (78). In addition, only one manufacturer provides an immunoassay kit for mouse anti-rubella immunoglobulin G (IgG) in the US (Alpha Diagnostic International). Therefore the negative results could also be due to the low sensitivity and specificity of our mouse anti-rubella IgG ELISA kit as previously described (79).

### Innate and adaptive antitumor immune response pathways are effectively activated during low IT doses of MMR immunovirotherapy

**Figure 3:**
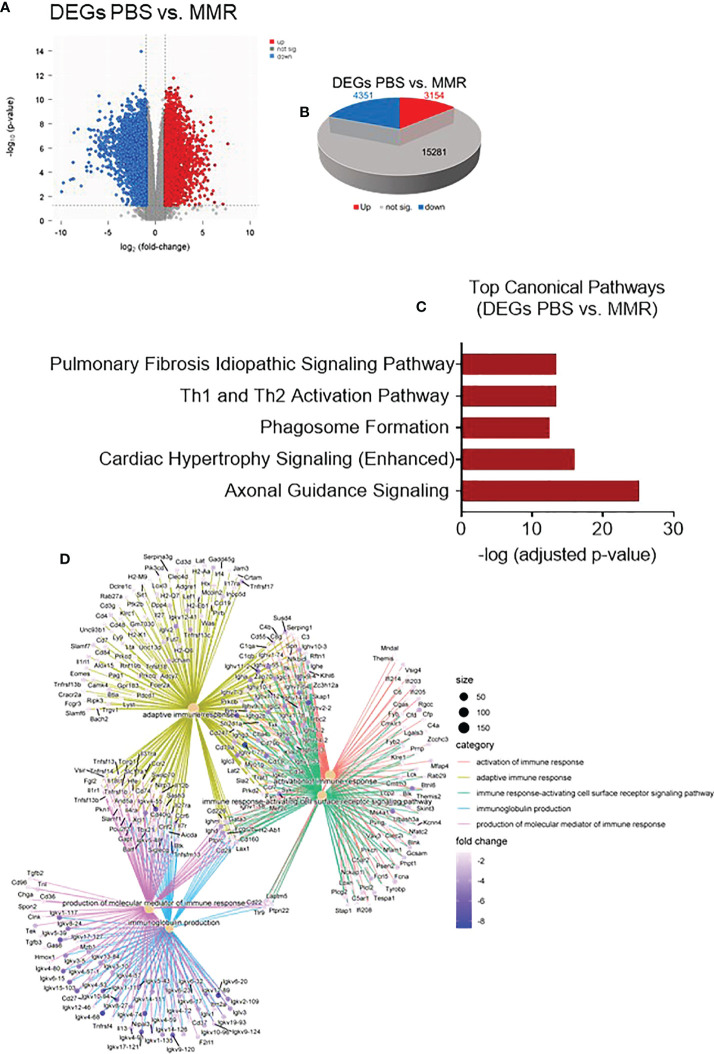
Transcriptomic analysis of Hepa 1-6 tumors treated with the MMR vaccine. **(A)** Volcano plot of mRNA expression differences for PBS vs. MMR. **(B)** 3-D Pie slices of the numbers of differentially expressed genes (DEGs) in PBS vs. MMR groups. DEGs were determined using the limma-voom method. A fold-change |logFC| ≥ 1 and false discovery rate (FDR) of 0.055 were used as a cutoff. The logFC was computed using the difference of the mean of log2(MMR) and mean of log2(PBS), that is, mean of log2(MMR) - mean of log2(PBS). **(C)** Graph showing top-scoring canonical pathways significantly enriched by treatment with MMR compared to PBS. **(D)** Gene ontology (GO) analysis using gseGO. Visualization of Gene Set Enrichment Analysis (GSEA) on the DEG between PBS controls and MMR groups.

On day 28, Hepa 1-6 tumors (n=3/group) were harvested and sectioned into two halves. One half was dissociated into a single-cell suspension (GentleMACS, Miltenyi) for flow cytometry, and the remaining half will be formalin-fixed and paraffin-embedded (FFPE). We extracted RNA from the FFPE tumors to analyze and compare the transcriptome of Hepa 1-6 tumors injected with PBS or MMR. Analysis of mRNA expression levels of these two groups using the limma-voom method (80) identified a storm of 3,154 upregulated and 4,351 downregulated genes (**Figures 3A, B** **)**; however, the expression of 15,281 genes was relatively unaffected. Based on this result, we performed Gene Set Enrichment Analysis (GSEA) on the DEGs between the PBS and MMR groups. We applied two functions (gseGO and gseKEGG) to identify the enriched terms in Gene Ontology (GO) and the Kyoto Encyclopedia of Genes and Genomes (KEGG) with a false discovery rate (FDR) value < 0.05. The immune-related top canonical pathways were Th1 and Th2 activation and phagosome pathways (**Figure 3C**). The top biological functions enriched by MMR were activation of the immune response, adaptive immune response, immune response-activating cells surface receptor signaling pathway, immunoglobulin production, and production of molecular mediator of immune response (**Figure 3D**). Furthermore, the KEGG analysis indicated that the differentially expressed genes were mainly involved in the immunoglobulin complex and circulating immunoglobulin complex, which is crucial in regulating antigen-mediated immune response (**Supplementary Figure 5**). These results suggest that mechanistically, MMR stimulates local antigen presentation, leading to activation and recruitment of cytotoxic effectors of innate and adaptive immunity, which are the hallmarks of a durable antitumor immune response (63, 81–85).

### Integrated transcriptomic and proteomic analysis revealed novel differentially expressed features associated with IT injections of MMR in murine HCC

**Figure 4:**
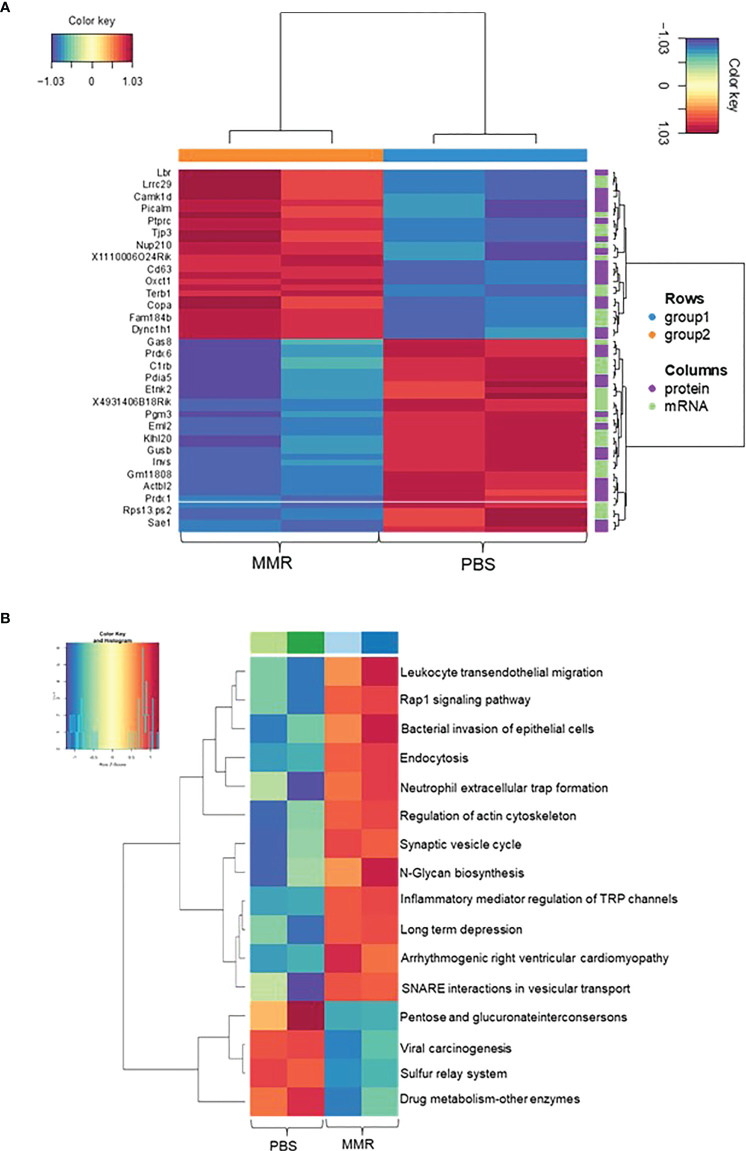
*Integration Analysis between transcriptome and proteome data of Hepa 1-6 tumors treated with MMR.* A MixOmics supervised analysis was carried out between DEGs and differentially expressed proteins (DEPs) based on Log2 fold change values. Log2 fold change of DEG × Log2 fold change of DEP >0 with a *P-value* of DEG and DEP <0.05 were considered associated DEGs/DEPs. **(A)** DEG/DEP expression heatmap of the 30 most up-regulated and down-regulated features in MMR vs. PBS groups. **(B)** Heatmap displaying enriched biological pathways filtered based on consistency between all MMR or PBS groups.

To investigate the changes between mRNA and protein expression levels following IT treatments with MMR, integration analysis of the transcriptomic and proteomic data (**Supplementary Figures 6A, B**) was performed. To do this, we first analyzed the proteomic data (mass spectrometry data) of differentially expressed proteins (DEPs), of which 134 were upregulated and 141 downregulated (**Supplementary Figures 6C, D**). Then, we applied a correlation analysis to examine the association between mRNA and protein expression levels in the two groups (PBS and MMR) of Hepa 1-6 tumors. A MixOmics supervised analysis was used to select the best features from the multi-omics data to differentiate mRNAs and corresponding expressed proteins between the PBS and MMR groups. In the associated DEGs/DEPs, we identified the top 30 enriched features that were significantly deregulated in the PBS group compared to the MMR group. (**Figure 4A**). Out of the DEGs intensities that correlate with their DEPs, the expression of the Dync1h1, COPA, CD63, PTPRC, NUP210, and OXCT1 was significantly upregulated in the MMR group compared to PBS. Conversely, the expression of ETNK2, EML2, and SAE1 was downregulated in the MMR vs. PBS groups. Some identified associated DEGs/DEPs have various and, at the same time, controversial functions in cancer. While downregulation of the dynein cytoplasmic 1 heavy chain (Dync1h1), an essential member of the intracellular transport of DNA damage proteins family, has been linked to poor prognosis and low survival in glioblastoma (86), its upregulation was shown to delay tumor proliferation in gastrointestinal tumor cells (87). Similarly, the coatomer subunit α (COPA) gene encodes the human homolog of the a-subunit coatomer protein complex involved in intracellular protein transport. Loss of COPA has also been associated with the high proliferation and invasiveness of HCC cells (88). Other features, such as CD63, were identified, which overexpression negatively regulated tumor invasiveness, including HCC (89). Furthermore, several of these genes and their protein products, such as PTPRC, NUP210, OXCT1, ETNK2, are known to be upregulated in tumors cells, including HCC (90–93). Others are involved in cellular senescence, apoptosis, angiogenesis, inflammation, and wound healing (LBR, EML2, SAE1) (94–97). Some of these genes (i.e., CAMK1D, TJP3, GAS8, and PDIA5) are associated with epithelial-mesenchymal transition (EMT), immune cells infiltration, and immune evasion (98, 99).

### IT administration of MMR correlates with activation of inflammatory immune response and cellular cytotoxicity pathways

Analysis of top KEGG pathways of the associated DEGs/DEPs using MOGSA highlighted an upregulation of Leukocyte transendothelial migration, endocytosis (phagocytosis), neutrophil extracellular traps signaling (cellular death) pathways, and downregulation of pathways like viral carcinogenesis and drug metabolism, suggesting an MMR-induced activation of inflammation, innate and adaptive immune responses (**Figure 4B**). Downregulation of other pathways such as hypoxia and reactive oxygen species, revealed by the MOGSA (100) analysis in MMR-treated tumors, also indicated activation of cellular metabolic arrest and apoptosis (**Supplementary Figure 7**). These results further validate our transcriptomic findings indicating that MMR can function as a cancer vaccine recruitment and activation of effectors of innate and adaptive antitumor immune response pathways.

## Discussion

Studies have shown that defects in interferon (IFN) pathways favor OV-mediated tumor-restricted oncolysis (101, 102). OVs can infect, replicate and lyse a wide range of mammalian tumor cells, leaving normal cells unaffected (7–13). In addition, virus-mediated oncolysis also provides needful conditions for priming antitumor immunity by activation of tumor-specific cytotoxic T cells (37, 54, 81–85, 103–107). To date, there are only two immunovirotherapies approved for use in human cancers, including the herpes simplex virus T-Vec and the modified adenovirus H101 (108–110) even though many other OVs have been evaluated clinically (14–17, 26, 111–114). Besides heterogeneity in clinical response, the cost-intensive nature of manufacturing of current OVs also make them far out of reach for most patients (20). The trivalent MMR vaccine effectively modulates durable immunity against measles, mumps, and rubella, making it one of the most successful human vaccines to date (30–35). Numerous preclinical and clinical studies have demonstrated the high immunogenicity and antitumor efficacy of vaccine lineages of measles and mumps (24–27). Based upon extensive data on the immunomodulatory properties of MMR, we evaluate the antitumor potential of its IT administrations in two preclinical mouse models.

Here, we show that IT immunotherapy with low doses of MMR lead to prominent tumor growth delay and improved survival in subcutaneous syngeneic hepatocellular carcinoma (HCC) and colorectal cancer (CRC) models. HCC disproportionally affects patients from disadvantaged backgrounds globally (38, 115), and the liver is one of the dominant metastatic sites for colorectal cancer cells (44, 45). Additionally, HCC is often localized at advanced stages (116), allowing intratumoral administrations of high concentrations of viral vectors while mitigating off-target effect and the impact of virus-neutralizing antibodies (NAbs) (117–119). Hence, why we chose to focus primarily on HCC and CRC in this study.

A plethora of studies have shown that the antitumor activity of OVs is predominantly due to the potent activation of tumor-specific T cells that recognize tumor antigens (82, 84, 85, 105, 107). In this study, we also show that a low dose of MMR can stimulate an immune response within the tumor, which, compared to control, is characterized by an increasing subset of CD8+ GranzymeB+ TILs and decreasing frequency of macrophage populations in murine HCC model. Previous works have pointed out that pre-existing immunity to OVs is detrimental to their oncolytic activity (36, 118, 120–122). Accordingly, strategies to overcome this limitation have been proposed with various degrees of success (109, 117, 118, 123). However, while we detected antibodies to measles and mumps viruses in the serum of MMR-treated mice, our ELISA kit failed to detect antibodies against rubella viruses.

So far, the preferred method of injecting OVs in patients with solid tumors is the IT route of administration (124). In addition, it has been shown that antibodies targeting measles hemaglutinin (H) protein can enhance its cellular uptake rather than inhibit viral entry (78). These results suggest that IT administration of low doses of MMR is safe and elicits, among other, humoral immune response and can modulate the tumor microenvironment (TME), inducing tumor growth delay and prolonging survival in animal models.

Currently, there is a scarcity of transcriptomic and proteomic data elucidating the antitumor mechanisms of OVs. Therefore, we employed an integrated proteogenomic pipeline to identify mRNA and corresponding protein intensities differentially expressed following IT injections with MMR or control in murine HCC tumors. Our data shows that following IT injection of MMR in murine tumors, significant proteogenomic changes occurred that coincided with modulation of the TME *via* activation of innate and adaptive immunity leading to tumor control and survival benefits. Studies have showed that measles, mumps, and rubella are exclusive human pathogens, thus poorly replicate in mouse tissues (46–49). Thus, to study measles and vesicular stomatitis virus pseudotyped with measles entry proteins, we and others have used a modified mouse model expressing the human measles Edmonton strain receptor complement regulatory protein (CD46) (47, 125). Hence, we performed an *in vitro* cell viability assay and amplified viral genes to investigate whether the tumor growth inhibition was also due to virus-induced oncolysis.

In contrast with these previous reports, we found that low doses of MMR can infect and lyse murine HCC cells *in vitro*, suggesting that the *in vivo* anti-cancer activity of MMR is multimodal *via* potential induction of cytolytic cell death and activation of immune responses within the tumor which led to tumor regression and extended survival in the model studied (51–54, 126). These findings should of course not be interpreted as MMR induced an oncolysis dependent or independent antitumor activity and be more a function of heterogeneity of individual viruses (measles, mumps, and rubella viruses) infectivity across specific mouse strains and tumor models. Therefore, using non-lytic or inactivated viruses in subsequent studies would be essential to understanding the virus-induced oncolysis in MMR-facilitated anti-cancer activity.

Besides, while oncolytic measles and mumps have demonstrated safety in laboratory animals and humans, relatively high viral titers are required to achieve the desired antitumor effect in humans (24–27, 37, 127). This can potentially be challenging in pediatric, older, pregnant patients and immunocompromised patients. Administration of low doses of attenuated oncolytic viruses has been widely used to reduce the risk of severe toxicity, environmental shedding, or reversion to the wild-type phenotype (55). In this study, we show that low IT doses of MMR can yield antitumor activity in mouse tumor models, thus could be a strategy for mitigating the concerns mentioned above (16, 37, 127, 128). Among other prominent causes of the antitumor activity of MMR at a low dose is that IT injection of the vaccine may elicit a polymorphic immune response against each virus (measles, mumps, and rubella viruses) as well as tumor antigens.

Our data indicate that IT immunotherapy with low doses of MMR correlated with induction of an immune response that led to tumor control, and survival benefit in murine tumor models. However, it does not determine if CD8+ T cells present within the tumor following IT injections of MMR are predominantly virus-specific CD8+ T cells or tumor-specific CD8+ T cells, or both (129–131). In addition, due to the use of vaccine-naïve mice, we could not provide insights into the influence of pre-existing NAbs to measles, mumps, and rubella viruses on the antitumor effect of MMR. We did not also determine if MMR can induce systemic antitumor immunity, which can effectively target and eliminate minimal residual disease (132, 133). Although pre-existing immunity to MMR could be detrimental to its antitumor activity by inhibiting viral infection of one or all three viruses, IT administration of OVs has been shown to alleviate issues with toxicity due to high doses the impact of antiviral immunity (mainly the effect of NAbs) on their oncolytic activity (134). Conversely, pre-existing immunity to OVs *via* the formation of immune complexes could trigger MHC-II presentation leading to tumor colonization of pre-existing cytotoxic T cells (i.e., CD8+ T cells, CD4+ T cells, etc.), amplifying their antitumor activity (135, 136). The type and stage of cancer, immune mechanisms, tumor microenvironment, timing, dosage, and route of administration are crucial for obtaining the desired therapeutic effect with OVs.

Albeit mechanistic questions remain to be addressed, this work provides evidence that IT immunotherapy based on a low dose of MMR can prime an immune response resulting in tumor regression. This study will enable the rational design of studies using MMR combined with low doses of other therapies, such as immune checkpoint inhibitors or radiation therapy, in early or late-stage solid tumors for possible additive or synergistic long-term responses in clinical settings (22).

Lastly, we have shown that therapeutic strategies that repurpose low-cost trivalent LAVs to activate antitumor immunity are achievable with MMR. But even so, to fully exploit the antitumor properties of MMR, future work should focus on elucidating whether this expanded population of CD8+ T cells within the treated tumors are tumor- or MMR-specific, or both. It will also be essential to understand to what extent (potency and durability) each virus (measles, mumps, and rubella) contribute to the MMR-enabled antitumor activity and if it can effectively target and eradicate minimal residual disease and distant metastases in experimental cancer models, then clinical studies.

## Materials and methods

### Cell lines

The murine hepatoma Hepa 1-6 (ATCC CRL-1830) and Vero (ATCC CCL-81) cell lines used in this study were purchased from ATCC and was cultured in Dulbecco’s modified eagle medium (DMEM) supplemented with 10% fetal bovine serum (FBS), 1% L-glutamine, and 1% penicillin/streptomycin. The murine colon adenocarcinoma cells MC-38 (Cat. # ENH204-FP; Kerafast) used in this study were obtained from Kerafast and cultured in DMEM with 10% FBS, 2 mM glutamine, 0.1 mM nonessential amino acids, 1 mM sodium pyruvate, 10 mM Hepes, 50 µg/mL gentamycin sulfate, and 1% penicillin/streptomycin. All cells were tested for mycoplasma and passaged in a tissue culture incubator at 37°C and 5% CO2.

### Preparation of the trivalent live attenuated measles, mumps, and rubella viruses (MMR)

The live attenuated MMR vaccine was purchased from the University of Arkansas for Medical Sciences (UAMS) pharmacy and contained attenuated live Edmonston measles, B level Jeryl Lynn mumps, and RA 27/3 Rubella viral strains. A single immunizing dose (single 500 µL vial) of the MMR vaccine delivers 1 × 10^3^, 1.25 × 10^3^, and 1 × 10^3^ median tissue culture infectious doses (TCID_50_) of measles, mumps, and rubella viruses. In this study, we used a 10-fold lower dose (1 × 10^2^, 1.25 × 10^2^, and 1 × 10^2^ TCID_50_ for each virus) compared to the immunizing dose. To prepare the vaccine for animal studies, lyophilized MMR vaccine powder vials were reconstituted and diluted with the provided diluents as recommended by the manufacturer (Merck).

#### Visualization of MMR-induced plaque formation in Vero cells

We used a low dose of MMR (1 x 10^2^ TCID_50_) to infect adherent Vero cells (2.5 × 10^5^ cells per well) in 6-well plates. Cells were incubated at 37°C until analysis. At 4 days post-infection, cells were fixed with 10% paraformaldehyde (PFA) and stained with 0.1% crystal violet to visualize virus-induced plaques in infected and mock-infected wells. We took pictures of representative areas in two MMR-infected wells and one mock infected well.

#### Cell viability assays

For all cytotoxicity assays (96-well format), 1.5 x 10^4^ cells were mock-infected or infected with MMR at the indicated TCID_50_ of 1000, 100, and 10 in serum-free Gibco Minimum Essential Media (Opti-MEM). Cell viability was determined using Cell Titer 96 AQueous One Solution Cell Proliferation Assay (Promega Corp, Madison, Wisconsin, USA). Data was generated as means of six replicates from two independent experiments +/- SEM.

#### Animal studies

Under a UAMS approved Animal Use Protocol, we conducted the *in vivo* evaluations described below.

### *In vivo* efficacy of the MMR vaccine in a syngeneic HCC and CC models

To evaluate the *in vivo* therapeutic efficacy of the MMR vaccine in subcutaneous mouse HCC and CC models, 1 × 10^6^ Hepa 1-6 (n=7/group) or MC38 (n=8/group) cells in 100 µL of cold RPMI were injected subcutaneously into the right flanks of immunocompetent female C57BL6/J mice, Jackson Laboratories, using 1 mL syringes. Mice were monitored weekly for palpable tumors or any changes in appearance or behavior. When average tumors reached a treatable size (80-120 mm^3^), mice were randomized into the respective study groups and dosed within 24 hours of randomization. On days 0, 7, and 14, mice were administered 50 µL IT injections containing PBS (PBS control groups) or 1 × 10^2^ TCID_50_ units (a 10-fold lower dose compared to the immunizing dose used in children) of MMR (MMR groups). Tumor volume and body weight were measured twice weekly following randomization and initiation of treatment using a digital caliper and balance. Tumor volume was calculated using the following equation: (longest diameter * shortest diameter2)/2 with a digital caliper. During the first week of treatment and after each injection, mice were monitored daily for signs of recovery for up to 72 hours. Mice were euthanized when body weight loss exceeded 20%, when tumor size was larger than 2,000 mm³, or for adverse effects of treatment.

### Analysis of tumor-infiltrating immune cells

Hepa 1-6 tumors (n= 6 for PBS, n=3 for MMR) were excised and dissociated using a mouse tumor dissociation kit (Miltenyi, CAT# 130-096-730) with a gentleMACS^™^ Octo Dissociator (Miltenyi) according to the manufacturer’s protocol. CD45^+^ cells were isolated with mouse CD45 (TIL) microbeads (Miltenyi). Cells were incubated with Fixable Viability Stain 510 for 15 minutes at 4°C followed by anti-Fc blocking reagent (Biolegend, Cat# 101320) for 10 minutes prior to surface staining. Cells were stained, followed by data acquisition with a BD LSRFortessa X-20 flow cytometer. All antibodies were used following the manufacturer’s recommendation. Fluorescence Minus One control was used for each independent experiment to establish gating. For intracellular staining of granzyme B, cells were stained using an intracellular staining kit (Miltenyi, Inside Stain Kit Cat# 130-090-477), and analysis was performed using FlowJo™ (TreeStar). Forward scatter and side scatter cytometry were used to exclude cell debris and doublets.

### Flow cytometry antibody analysis

The following antibodies were used for flow cytometry analysis: CD45-FITC (Cat. # 553079; BD Biosciences), CD3-BUV395 (Cat. # 563565; BD Biosciences), CD4-BUV737 (Cat. # 612761; BD Biosciences), CD8-Percp-Cy5.5 (Cat. # 45-0081-82; eBioscience), CD44-BV711 (Cat. # 103057; Biolegend), CD335-PE/Dazzle594 (Cat. #137630; Biolegend), PD-1-PE (Cat. # 551892; BD Biosciences), Ki67*-BV605 (Cat. # 652413; Biolegend), Granzyme B*-APC (Cat. # 366408; Biolegend), IFN-γ*-BV421 (Cat. # 563376; BD Biosciences), CD11b-PE-Cy7 (Cat. # 101216; Biolegend), F4/80-BV510 (Cat. # 123135; Biolegend), CD206-AF700 (Cat. # 141734; Biolegend), I-A/I-E-BV786 (Cat. # 743875; BD Biosciences), and L/D-efluor780 (Cat. # 65-0865-18; eBioscience).

### Proteomic analysis of HCC formalin-fixed paraffin-embedded tissues

Fixed Hepa 1-6 tumors were dehydrated using an increasing ethanol concentration and embedded into paraffin to become formalin-fixed paraffin-embedded (FFPE) blocks as previously described (137). Following deparaffinization of FFPE samples with xylene and tissue lysis in sodium dodecyl sulfate, total protein was reduced, alkylated, and digested using filter-aided sample preparation (138) with sequencing grade modified porcine trypsin (Promega). Tryptic peptides were separated by reverse-phase XSelect CSH C18 2.5 µm resin (Waters) on an in-line 150 × 0.075 mm column using an UltiMate 3000 RSLCnano system (Thermo). Peptides were eluted using a 60 min gradient from 98:2 to 65:35 (buffer A, 0.1% formic acid, 0.5% acetonitrile: buffer B, 0.1% formic acid, 99.9% acetonitrile) ratio. Eluted peptides were ionized by electrospray (2.4 kV) followed by mass spectrometric (MS) analysis on an Orbitrap Exploris 480 mass spectrometer (Thermo). MS data were acquired using a Fourier transform MS (FTMS) analyzer in profile mode at a resolution of 120,000 over a range of 375 to 1500 m/z. Following HCD activation, MS/MS data were acquired using the FTMS analyzer in centroid mode at a resolution of 15,000 and normal mass range with normalized collision energy of 30%. Proteins were identified by database search using MaxQuant (Max Planck Institute) label-free quantification with a parent ion tolerance of 2.5 ppm and a fragment ion tolerance of 20 ppm. Scaffold Q+S (Proteome Software) was used to verify MS/MS-based peptide and protein identifications. Protein identifications were accepted if they could be established with less than 1.0% false discovery and contained at least two identified peptides. Protein probabilities were assigned by the Protein Prophet algorithm (139).

### RNA-seq data analysis of murine tumors

Hepa 1-6 FFPE scrolls were processed for DNA and RNA extraction using a Quick-DNA/RNA FFPE Miniprep Kit with on-column DNase digestion for the RNA preps (Cat. # R1009; Zymo Research). RNA was assessed for mass concentration using the Qubit RNA Broad Range Assay Kit (Cat. # Q10211; Invitrogen) with a Qubit 4 fluorometer (Cat. # Q33238; Invitrogen). RNA quality was assessed with a Standard Sensitivity RNA Analysis Kit (Cat. # DNF-471-0500; Agilent) on a Fragment Analyzer System (Cat. # M5310AA; Agilent). Sequencing libraries were prepared using TruSeq Stranded Total RNA Library Prep Gold (Cat. # 20020599; Illumina). RNA DV200 scores were used to determine fragmentation times. Libraries were assessed for mass concentration using a Qubit 1X dsDNA HS Assay Kit (Cat. # Q33231; Invitrogen) with a Qubit 4 fluorometer (Cat. # Q33238; Invitrogen). Library fragment size was assessed with a High Sensitivity NGS Fragment Analysis Kit (Cat. # DNF-474-0500; Agilent) on a Fragment Analyzer System (Cat. # M5310AA; Agilent). Libraries were functionally validated with a KAPA Universal Library Quantification Kit (Cat. # 07960140001; Roche). Sequencing was performed to generate paired-end reads (2 × 100 bp) with a 200-cycle S1 flow cell on a NovaSeq 6000 sequencing system (Illumina).

### Bioinformatics analysis

We examined the mRNA and protein expression profiles of Hepa 1-6 tumors treated with PBS or MMR. Three replicates were used to analyze each of the untreated (PBS) and treated (MMR) groups. The tumor samples were sequenced on an NGS platform. The files containing the sequencing reads (FASTQ) were then tested for quality control (QC) using MultiQC (140). The Cutadapt tool trims the Illumina adapter and low-quality bases at the end. After the quality control, the reads were aligned to a mouse reference genome (mm10/GRCm38) with the HISAT2 aligner (141), followed by counting reads mapped to RefSeq genes with feature counts. We generated the count matrix from the sequence reads using HTSeq-count (142). Genes with low counts across the samples affect the false discovery rate, thus reducing the power to detect differentially expressed genes; thus, before identifying differentially expressed genes, we filtered out genes with low expression utilizing a module in the limma-voom tool (80). Then, we normalized the counts by using TMM normalization (143), a weighted trimmed mean of the log expression proportions used to scale the counts of the samples. Finally, we fitted a linear model in limma to determine differentially expressed genes and expressed data as mean ± standard error of the mean. All *p* values were corrected for multiple comparisons using Benjamini-Hochberg FDR adjustment. After identifying differentially expressed genes, enriched pathways were performed using the Ingenuity Pathway Analyses tool to gain biological insights. The statistical difference between groups was assessed using the nonparametric Mann-Whitney U test R module.

### Integration of transcriptomics and proteomics

The limma-normalized transcript expression levels and the normalized protein intensities were integrated using two independent methods. Firstly, the mixOmics package (Omics Data Integration Project R package, version 6.1.1) was implemented to generate **Figure 4A** and **Supplementary Figure 5** as previously described (144). Secondly, the MOGSA package was used to generate Fig 4b and Supplementary Figs. 7 (100).

### Murine immunoglobulin differentiation following treatment with MMR

Serum samples were obtained from infected tumor-bearing mice from whole blood collected in BD Microtainer tubes. Mouse anti-measles IgG (Cat. # 530-130-MMG; Alpha Diagnostic), mouse anti-mumps IgG (Cat. #520-130-MMG; Alpha Diagnostic), and mouse anti-rubella IgG (Cat. # 510-120-MRG; Alpha Diagnostic) were used to determine the levels of anti-measles, anti-mumps, and anti-rubella antibodies in the serum samples by ELISA according to the manufacturer’s instructions.

### Blood chemistry and cytokines

Blood chemistry analysis was performed with an Abaxis Piccolo Xpress chemistry analyzer (Abaxis) to assess liver toxicity (i.e., aspartate transaminase, alkaline phosphatase, albumin), nephrotoxicity (i.e., creatinine, blood urea nitrogen), and serum electrolytes. Murine type I interferon-beta assay was performed using Mouse IFN beta SimpleStep ELISA^®^ Kit (Cat. # ab252363; Abcam).

### TUNEL assay immunohistochemistry

Tumor tissue sections were subjected to the terminal deoxynucleotidyl transferase deoxyuridine triphosphate nick-end labeling (TUNEL) assay using the *In Situ* Cell Death Detection Kit (Roche Diagnostics, Indianapolis, IN) according to the manufacturer’s protocol. After staining, cells were counterstained with 4’,6-diamidino-2-phenylindol (DAPI) to visualize cell nuclei, mounted under cover slips with Prolong^®^ Antifade kit (Invitrogen, Carlsbad, CA) and acquired using the Olympus IX-81 inverted microscope (Olympus America, Center Valley, PA) equipped with Hamamatsu ORCA-ER monochrome camera (Hamamatsu Photonics K.K., Hamamatsu City, Japan). Image analysis was performed using SlideBook 6.2 software. For quantification, 10 independent fields of view were collected per each well (each n) and mean optical density (MOD) or area of colocalization in pixels were recorded for Fluorescein (TUNEL) channel.

### Quantitative reverse transcriptase polymerase chain reaction

Quantitative reverse transcriptase polymerase chain reaction (qPCR) primers specific to measles nucleocapsid gene, mumps matrix gene, and rubella (envelope glycoprotein E1) were designed and synthesized using the PrimeTime™ qPCR program (Integrated DNA Technologies [IDT], USA). First, a reverse transcription-polymerase chain reaction was performed to generate cDNA using the High-Capacity cDNA Reverse Transcription Kit (Cat. # 4374966; Applied Biosystems). Followed by amplification of the cDNA to detect the presence of measles, mumps and rubella using the GeneAmp^®^ Fast PCR Master Mix (2X) (Cat. # 4359187; Applied Biosystems). Cycle conditions were 10 minutes at 95°C followed by 45 cycles of 45 s at 93°C, 1 minute at 72°C and 5 minutes at 72°C. Finally, qPCR reactions were performed on an Applied Biosystems StepOnePlus™ Real-Time PCR System (Applied Biosystems) using the PowerUp™ SYBR ™ Green Master Mix (Cat. # A25742; Applied Biosystems). Cycle conditions were 10 minutes at 94°C followed by 40 cycles of 10 s at 94°C and 1 minute at 60°C. Cycle threshold extraction was performed using the iCycler IQ software (version 3, Biorad). The following primers were used: forward primer (measles)- 5’CCT CAA TTA CCA CTC GAT CCA G 3’, reverse primer (measles)-5’ TTA GTG CCC CTG TTA GTT TGG 3’; forward (mumps) 5’ TCA AGC CAG AAC AAG CCT AG 3’, reverse (mumps)- 5’ TTG ATA ACA GGT CCA GGT GC 3’ and forward (rubella) 5’ TTG AAC CTG CCT TCG GAC 3’, reverse (rubella)-5’ CCT GGT CTC TGT ATG GAA CTT G 3’.

### Statistical analysis

All values were expressed as the mean ± standard error of mean, and the results were analyzed by one-way analysis of variance followed by the Tukey test or Benjamini-Hochberg FDR adjustment for multiple comparisons and *t* test to compare group means. Kaplan-Meier method for survival, using statistical software in GraphPad Prism, version 8 (GraphPad Software). A *p* value less than 0.05 was considered statistically significant.

## Data availability statement

The original contributions presented in the study are included in the article/**Supplementary Material**. Further inquiries can be directed to the corresponding author. The DNA methylation and RNA sequencing data are freely available via GEOGSE199131, and the proteomics data are available via the ProteomeXchange PXD031295.

## Ethics statement

The animal study was reviewed and approved by UAMS ethics committee.

## Author contributions

AB, MB, MJC, and BN contributed to study concept and design, data acquisition, data analysis, data interpretation, and manuscript drafting. YZ, MG, MZT, MAT, ALG, CS, JC, CD, OB, AS, SP, and TK contributed to data acquisition, data analysis, data interpretation, drafting and critical revision of the manuscript. MC contributed to drafting and critical revision of the manuscript. CW, DA, AG, and SB contributed to bioinformatic analysis. All authors approved the final, submitted version of the manuscript.

## Funding

This work was supported by the National Institute of Health (NIH) through a National Cancer Institute (NCI) grant (CA234324 to BN); and start-up funds from the Winthrop P. Rockefeller Cancer Institute to BN. The UAMS Bioinformatics Core Facility is supported by the Winthrop P. Rockefeller Cancer Institute and NIH/NIGMS grant P20GM121293. The IDeA National Resource for Quantitative Proteomics is supported by NIGMS grant R24GM137786.

## Acknowledgments

We thank the personnel of the DNA Damage and Toxicology, Proteomics, Genomics, and Bioinformatic Cores at the University of Arkansas for Medical Sciences (UAMS) for their assistance during these studies.

## Conflict of interest

The authors declare that the research was conducted in the absence of any commercial or financial relationships that could be construed as a potential conflict of interest.

## Publisher’s note

All claims expressed in this article are solely those of the authors and do not necessarily represent those of their affiliated organizations, or those of the publisher, the editors and the reviewers. Any product that may be evaluated in this article, or claim that may be made by its manufacturer, is not guaranteed or endorsed by the publisher.

## Author disclaimer

Its contents are solely the responsibility of the authors and do not necessarily represent the official views of the NIH.

## Glossary

**Table:**

ALP	alkaline phosphatase
ALT	alanine aminotransferase
Anti-Measles	anti-measles fusion and hemagglutinin antibody
Anti-Mumps	anti-mumps fusion and hemagglutinin antibody
Anti-Rubella	anti-rubella envelope glycoprotein E
AST	aspartate aminotransferase
ATCC	American Tissue Culture Collection
CTLs	cytotoxic T lymphocytes
CRC	Colorectal cancer
DMEM	Dulbecco modified eagle medium
DPBS	dulbecco phosphate-buffered saline
ELISA	enzyme-linked immunosorbent assay
G-MDSCs	granulocytic myeloid-derived suppressor cells
GzmB	granzyme B;
h	human
HCC	hepatocellular carcinoma
LAV	live attenuated viral vaccine
MMR	live attenuated measles
mumps	and rubella virus vaccine
MOI	multiplicity of infection
PBS	phosphate-buffered saline
PD-1	programmed cell death protein 1
PE	phycoerythrin
RNA	ribonucleic acid
TAMs	tumor-associated macrophages
IT	intratumoral.
