# Supplementary material for: Repurposing live attenuated trivalent MMR vaccine as cost-effective cancer immunotherapy
Source: Front Oncol. 2022 Nov 9;12:1042250. doi: 10.3389/fonc.2022.1042250 (PMC9706410; doi:10.3389/fonc.2022.1042250)
Supplement: Supplementary file 1 [file Presentation_1.pptx]

## Slide 1
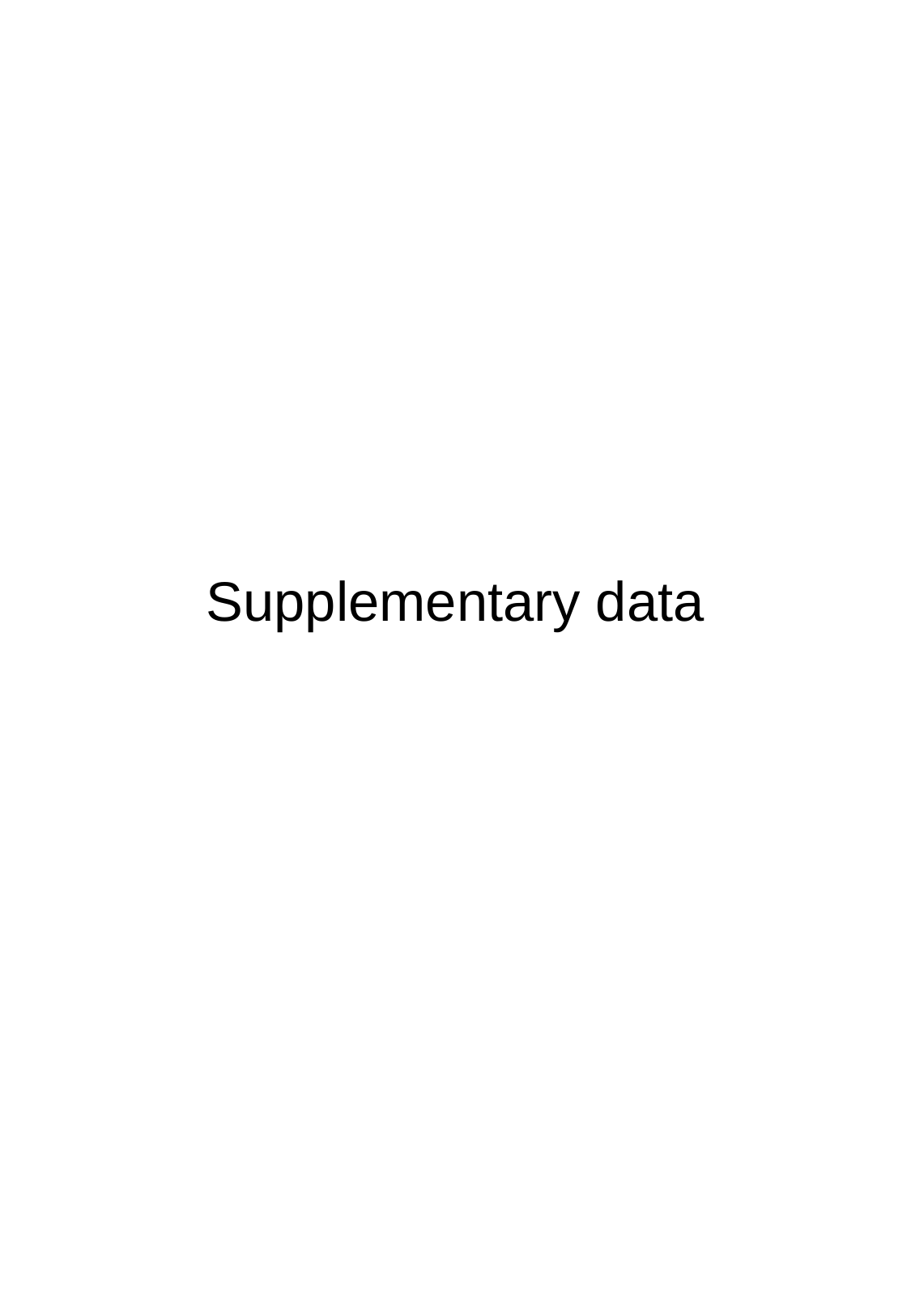

# Supplementary data

## Slide 2
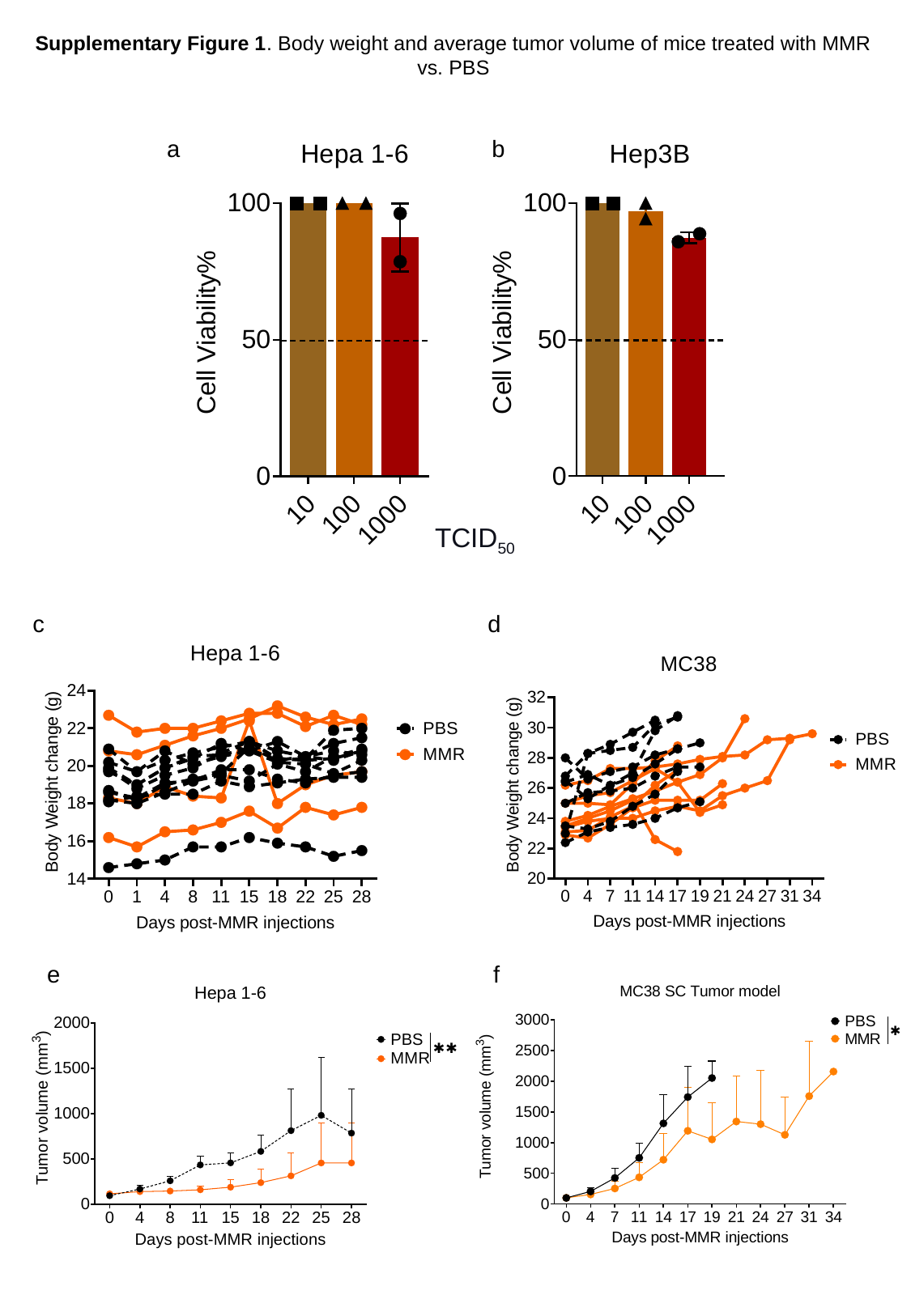

Supplementary Figure 1. Body weight and average tumor volume of mice treated with MMR vs. PBS
b
TCID50
a
d
c
e
f

## Slide 3
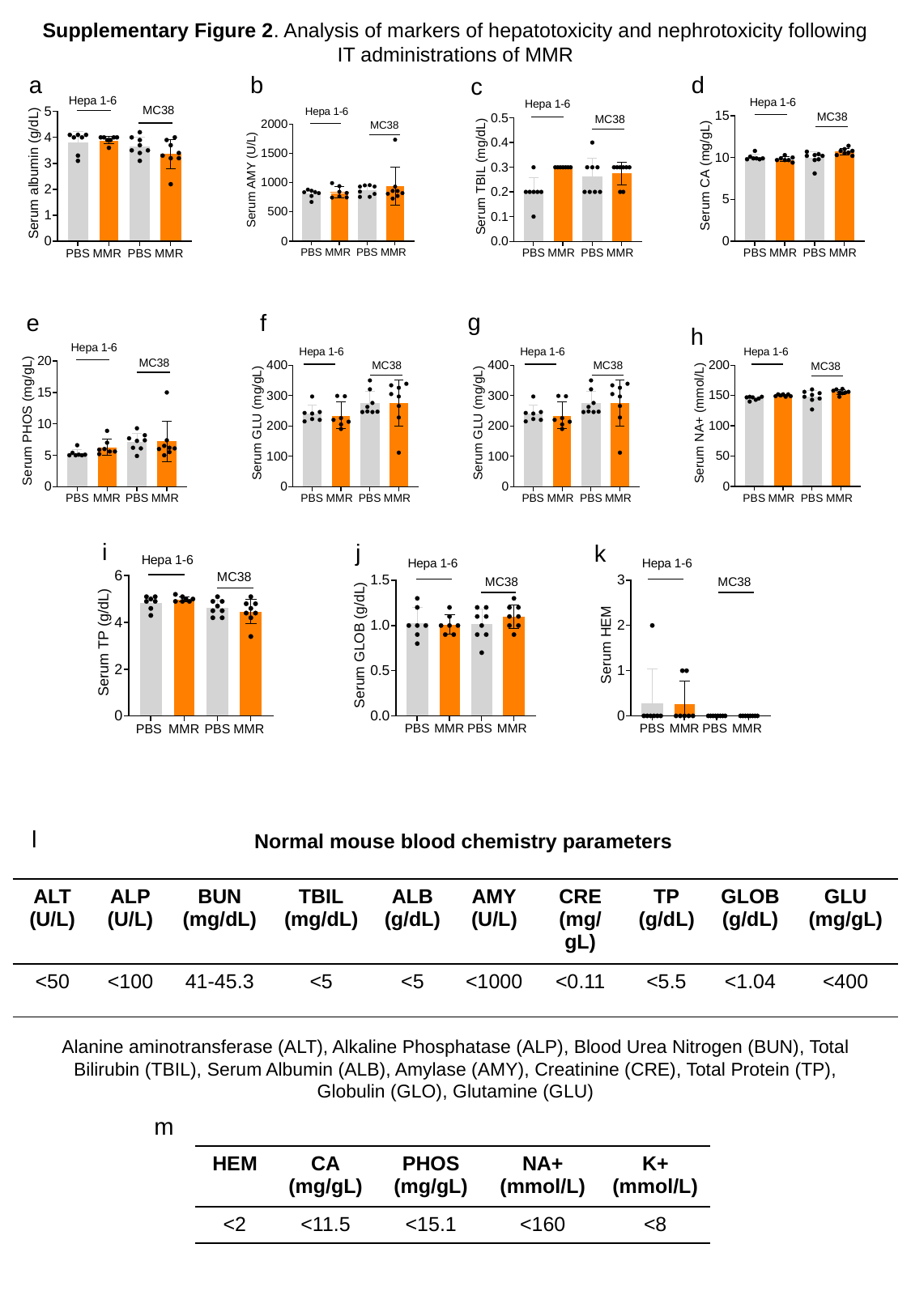

Supplementary Figure 2. Analysis of markers of hepatotoxicity and nephrotoxicity following IT administrations of MMR
a
b
d
c
g
e
f
h
i
j
k
l
Normal mouse blood chemistry parameters
| ALT (U/L) | ALP (U/L) | BUN (mg/dL) | TBIL (mg/dL) | ALB (g/dL) | AMY (U/L) | CRE (mg/gL) | TP (g/dL) | GLOB (g/dL) | GLU (mg/gL) |
| --- | --- | --- | --- | --- | --- | --- | --- | --- | --- |
| <50 | <100 | 41-45.3 | <5 | <5 | <1000 | <0.11 | <5.5 | <1.04 | <400 |
Alanine aminotransferase (ALT), Alkaline Phosphatase (ALP), Blood Urea Nitrogen (BUN), Total Bilirubin (TBIL), Serum Albumin (ALB), Amylase (AMY), Creatinine (CRE), Total Protein (TP), Globulin (GLO), Glutamine (GLU)
m
| HEM | CA (mg/gL) | PHOS (mg/gL) | NA+ (mmol/L) | K+ (mmol/L) |
| --- | --- | --- | --- | --- |
| <2 | <11.5 | <15.1 | <160 | <8 |
hem protein (HEM), Calcium (CA), Phosphate (PHOS), Sodium (NA+), Potassium (K+)

## Slide 4
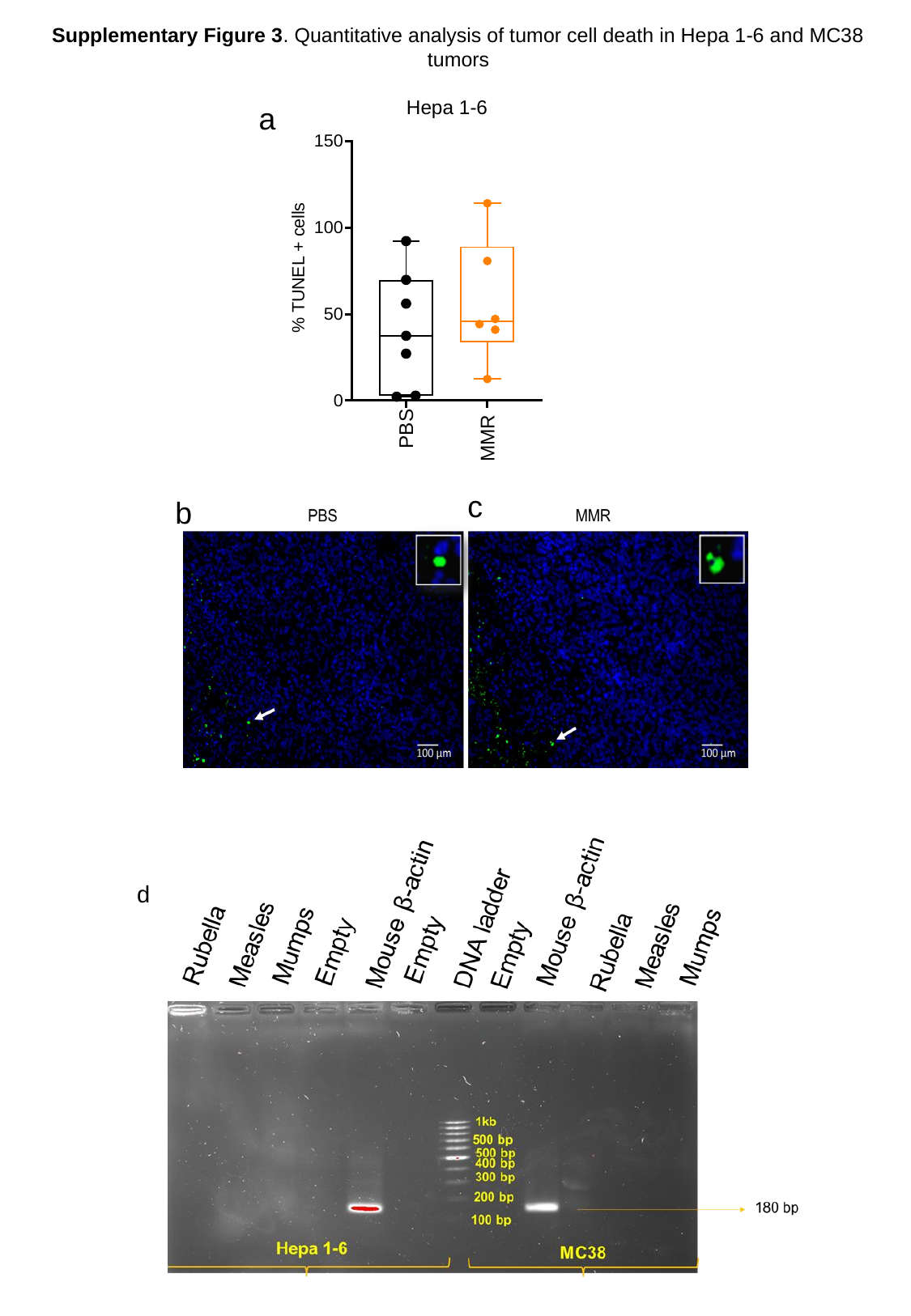

Supplementary Figure 3. Quantitative analysis of tumor cell death in Hepa 1-6 and MC38 tumors
a
c
b
d

## Slide 5
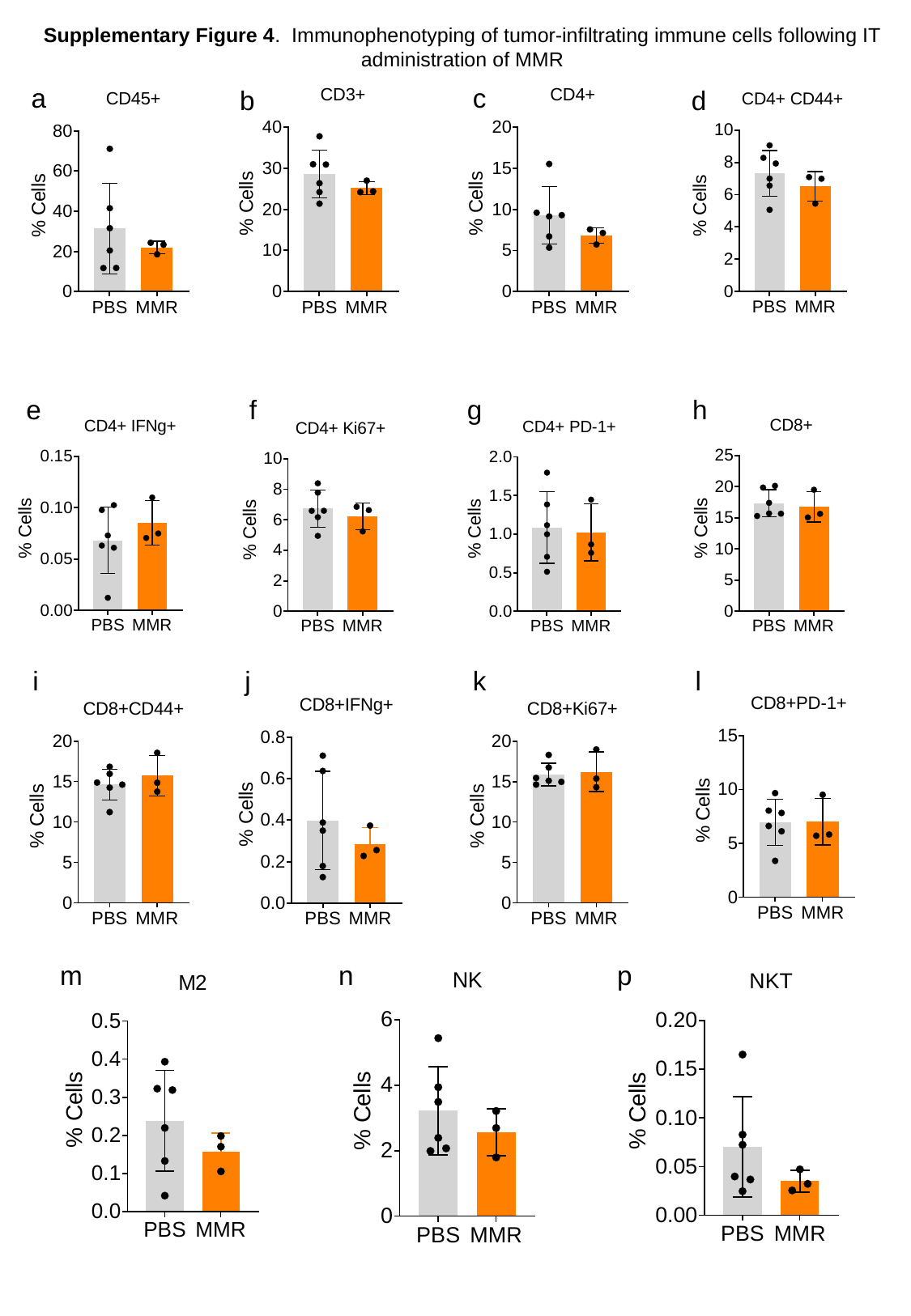

Supplementary Figure 4. Immunophenotyping of tumor-infiltrating immune cells following IT administration of MMR
a
c
b
d
g
h
e
f
i
j
l
k
m
n
p

## Slide 6
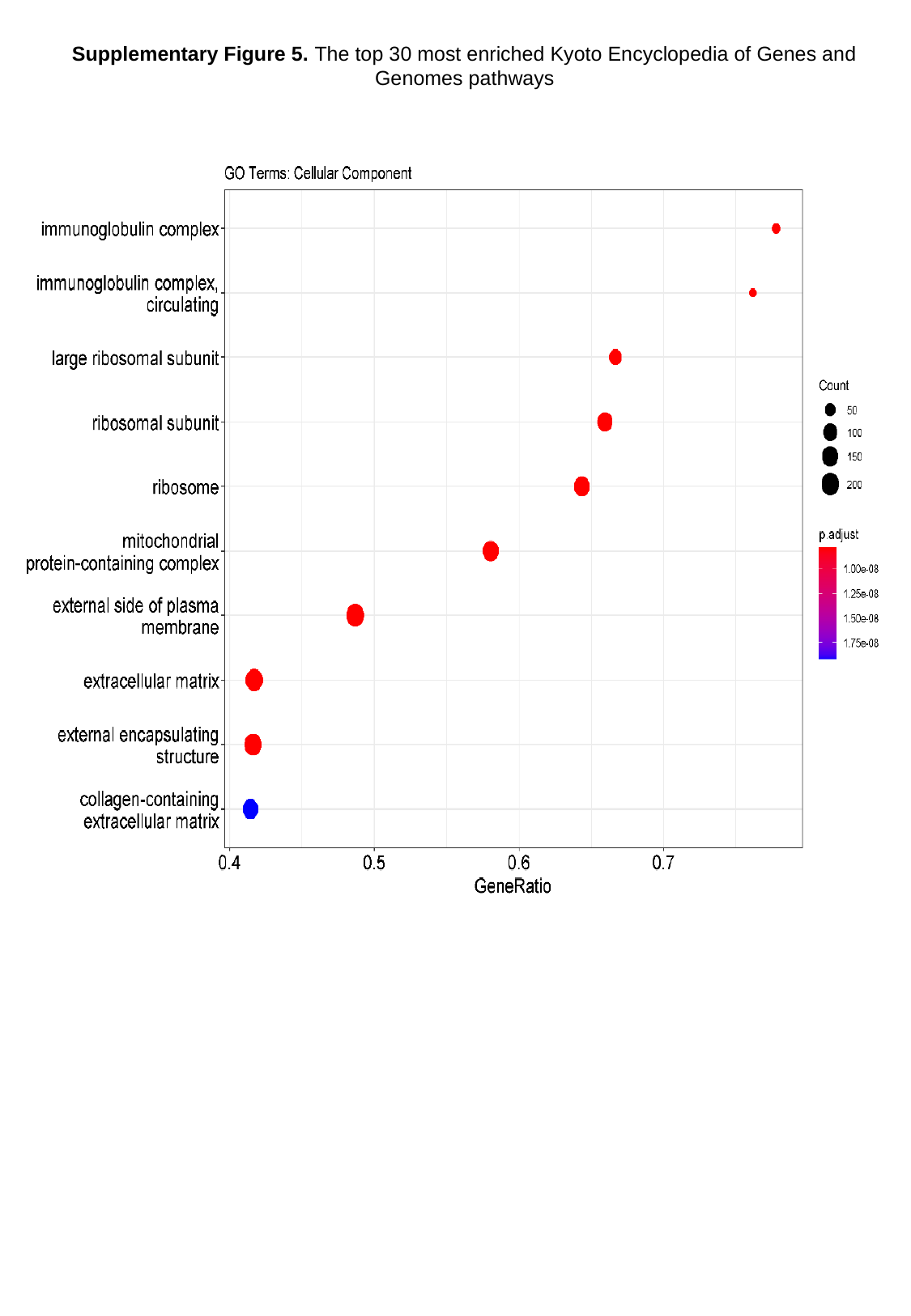

Supplementary Figure 5. The top 30 most enriched Kyoto Encyclopedia of Genes and Genomes pathways

## Slide 7
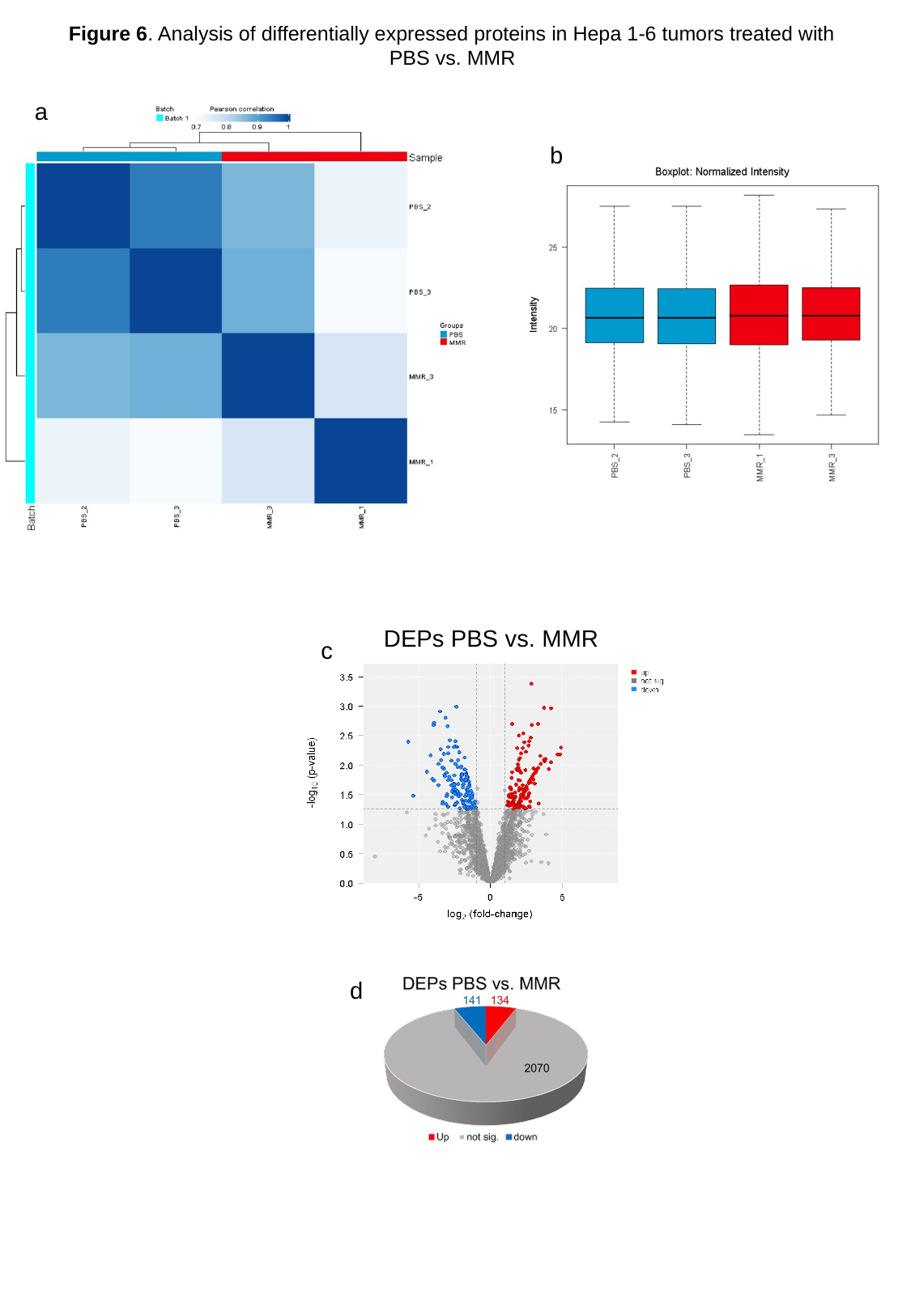

Figure 6. Analysis of differentially expressed proteins in Hepa 1-6 tumors treated with PBS vs. MMR
a
b
DEPs PBS vs. MMR
c
d

## Slide 8
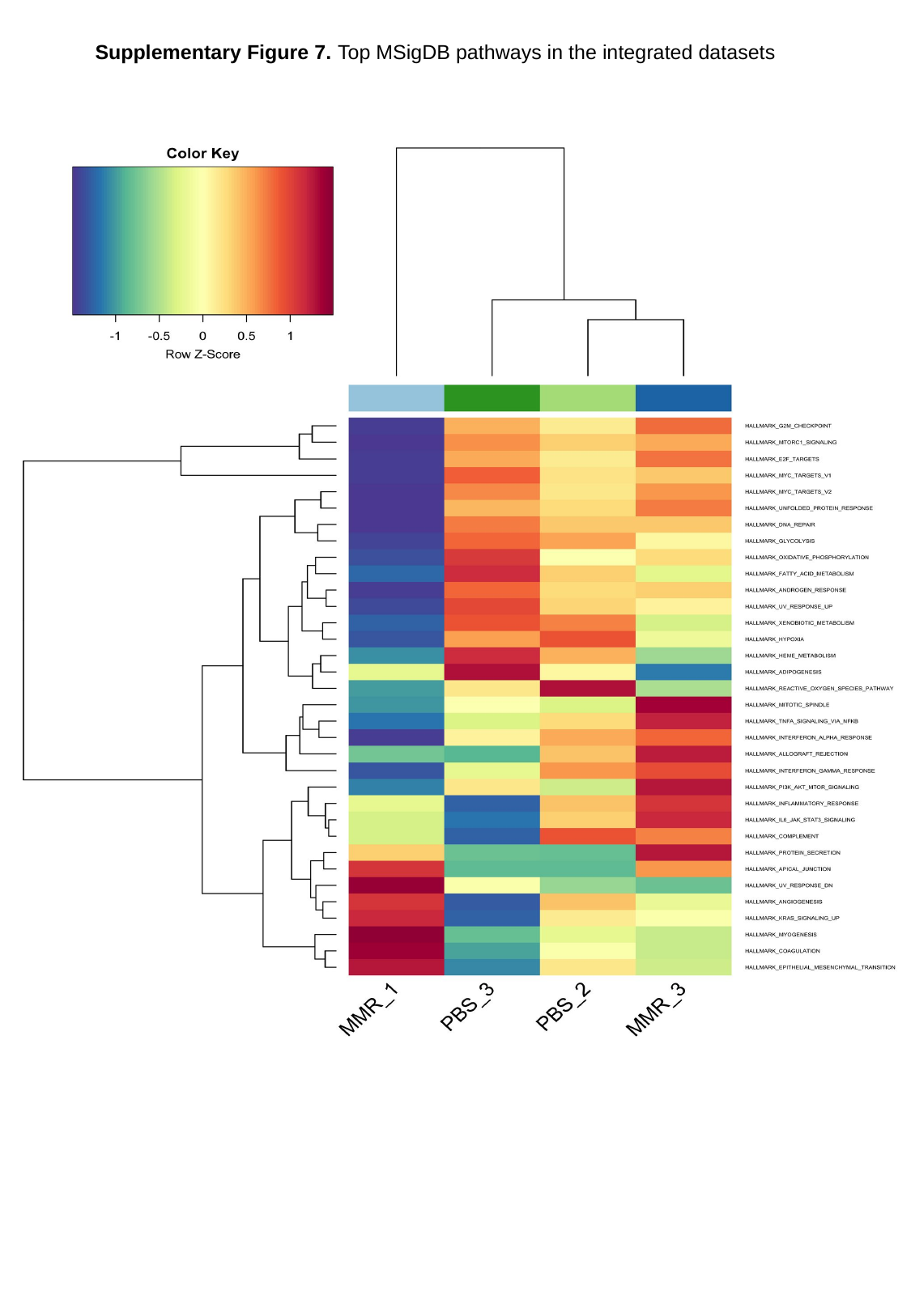

Supplementary Figure 7. Top MSigDB pathways in the integrated datasets

## Slide 9
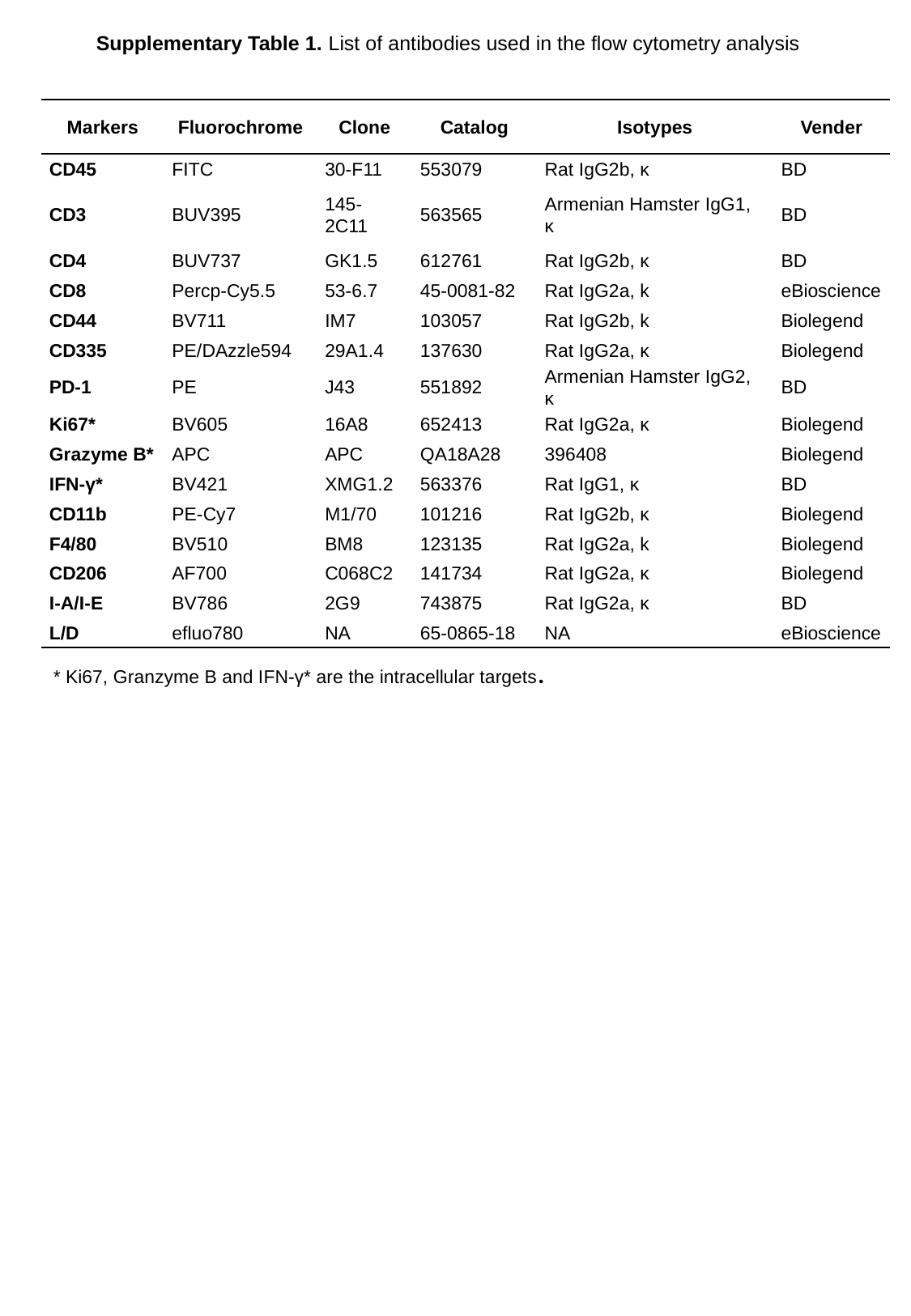

Supplementary Table 1. List of antibodies used in the flow cytometry analysis
| Markers | Fluorochrome | Clone | Catalog | Isotypes | Vender |
| --- | --- | --- | --- | --- | --- |
| CD45 | FITC | 30-F11 | 553079 | Rat IgG2b, κ | BD |
| CD3 | BUV395 | 145-2C11 | 563565 | Armenian Hamster IgG1, κ | BD |
| CD4 | BUV737 | GK1.5 | 612761 | Rat IgG2b, κ | BD |
| CD8 | Percp-Cy5.5 | 53-6.7 | 45-0081-82 | Rat IgG2a, k | eBioscience |
| CD44 | BV711 | IM7 | 103057 | Rat IgG2b, k | Biolegend |
| CD335 | PE/DAzzle594 | 29A1.4 | 137630 | Rat IgG2a, κ | Biolegend |
| PD-1 | PE | J43 | 551892 | Armenian Hamster IgG2, κ | BD |
| Ki67\* | BV605 | 16A8 | 652413 | Rat IgG2a, κ | Biolegend |
| Grazyme B\* | APC | APC | QA18A28 | 396408 | Biolegend |
| IFN-γ\* | BV421 | XMG1.2 | 563376 | Rat IgG1, κ | BD |
| CD11b | PE-Cy7 | M1/70 | 101216 | Rat IgG2b, κ | Biolegend |
| F4/80 | BV510 | BM8 | 123135 | Rat IgG2a, k | Biolegend |
| CD206 | AF700 | C068C2 | 141734 | Rat IgG2a, κ | Biolegend |
| I-A/I-E | BV786 | 2G9 | 743875 | Rat IgG2a, κ | BD |
| L/D | efluo780 | NA | 65-0865-18 | NA | eBioscience |
* Ki67, Granzyme B and IFN-γ* are the intracellular targets.
