# Supplementary material for: Repurposing live attenuated trivalent MMR vaccine as cost-effective cancer immunotherapy
Source: Front Oncol. 2022 Nov 9;12:1042250. doi: 10.3389/fonc.2022.1042250 (PMC9706410; doi:10.3389/fonc.2022.1042250)
Supplement: Supplementary file 2 [file DataSheet_1.pdf]

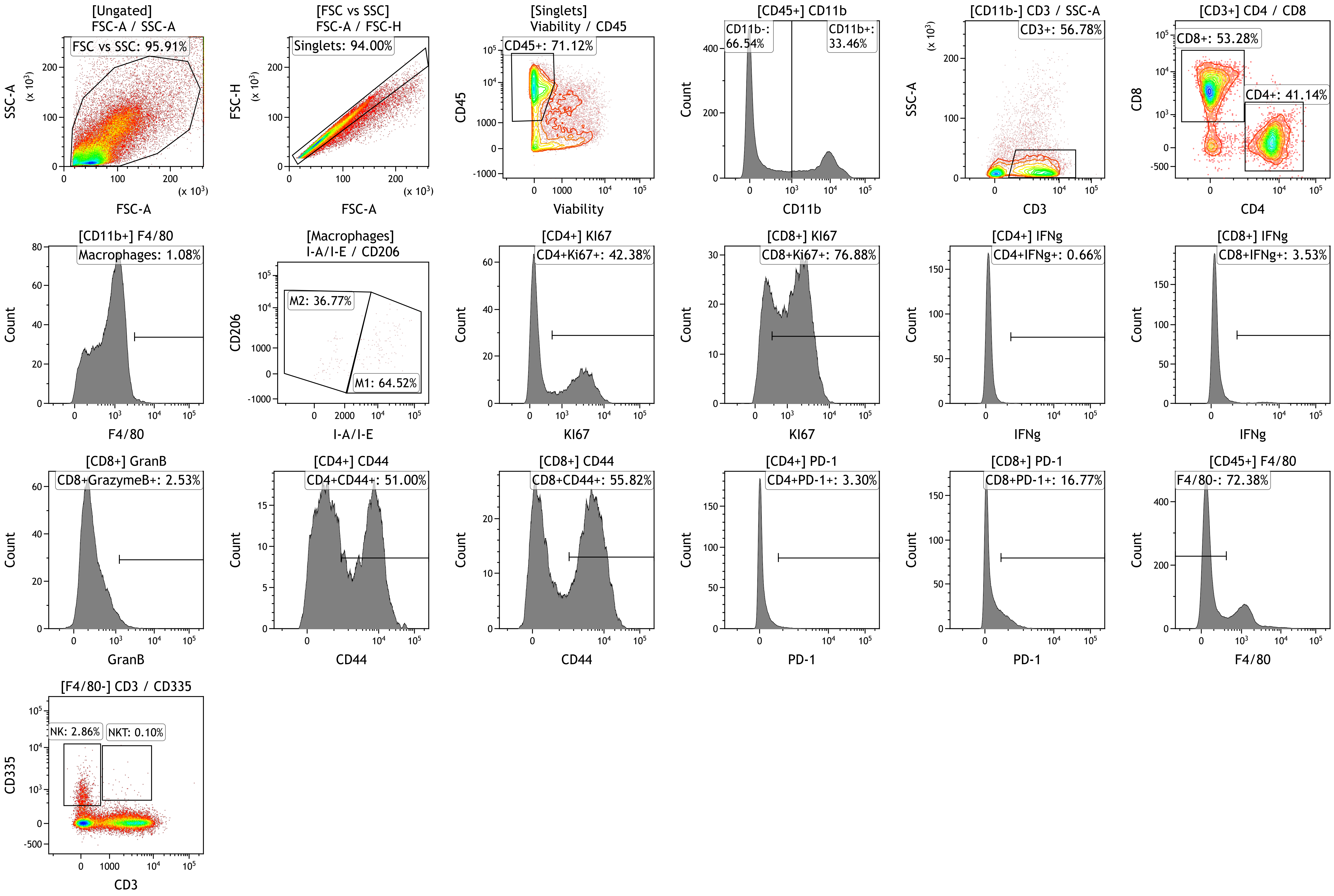

Gate	Number	%Gated
All	67,125	100.00
FSC vs SSC	64,380	95.91
Singlets	60,520	94.00
CD45+	43,040	71.12
CD11b-	28,640	66.54
CD3+	16,262	56.78
CD4+	6,690	41.14
CD4+CD44+	3,412	51.00
CD4+IFNg+	44	0.66
CD4+Ki67+	2,835	42.38
CD4+PD-1+	221	3.30
CD8+	8,665	53.28
CD8+CD44+	4,837	55.82
CD8+GrazymeB+	219	2.53
CD8+IFNg+	306	3.53
CD8+Ki67+	6,662	76.88
CD8+PD-1+	1,453	16.77
CD11b+	14,400	33.46
Macrophages	155	1.08
M1	100	64.52
M2	57	36.77
F4/80-	31,154	72.38
NK	891	2.86
NKT	32	0.10

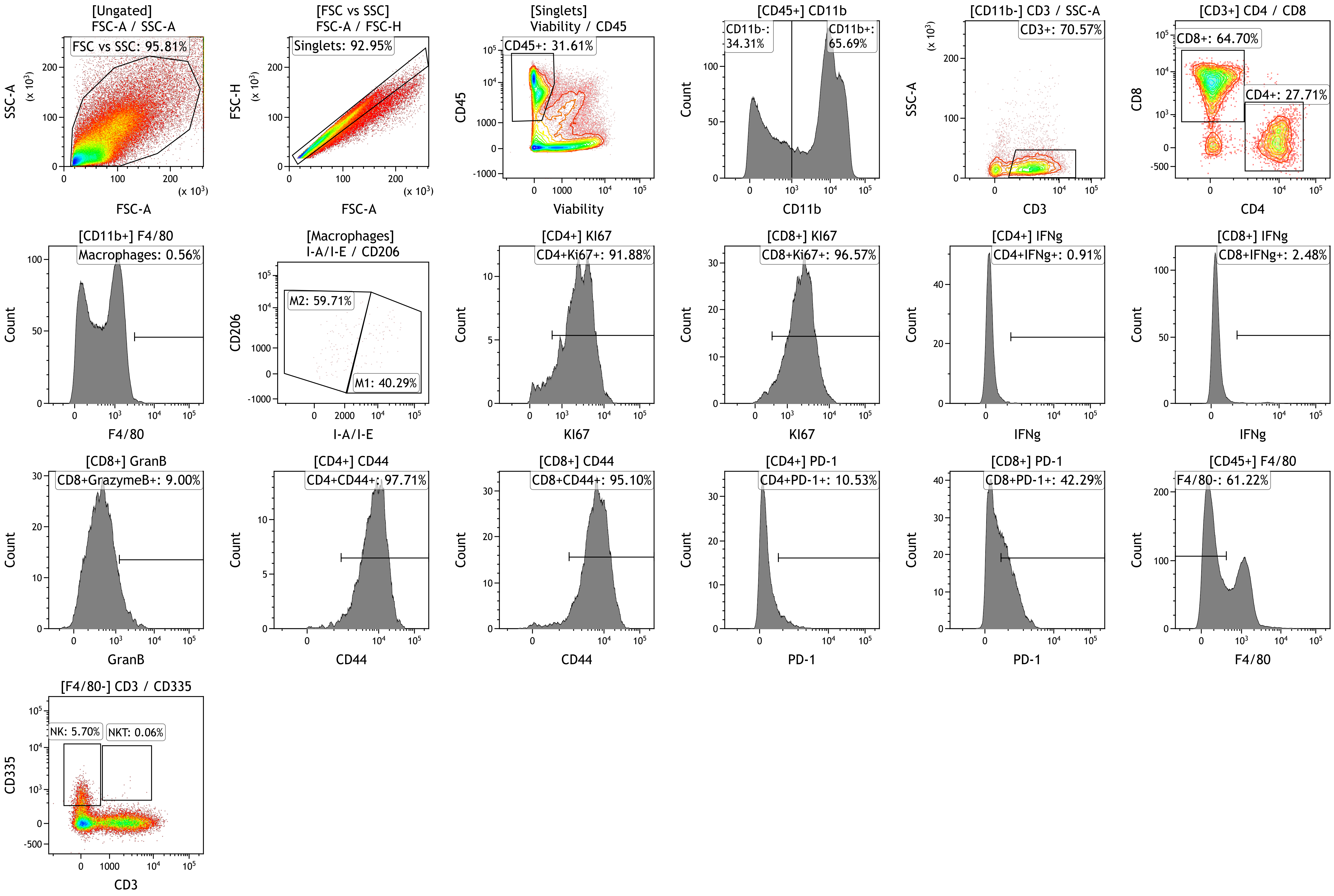

Gate	Number	%Gated
All	134,237	100.00
FSC vs SSC	128,610	95.81
Singlets	119,542	92.95
CD45+	37,792	31.61
CD11b-	12,968	34.31
CD3+	9,151	70.57
CD4+	2,536	27.71
CD4+CD44+	2,478	97.71
CD4+IFNg+	23	0.91
CD4+Ki67+	2,330	91.88
CD4+PD-1+	267	10.53
CD8+	5,921	64.70
CD8+CD44+	5,631	95.10
CD8+GrazymeB+	533	9.00
CD8+IFNg+	147	2.48
CD8+Ki67+	5,718	96.57
CD8+PD-1+	2,504	42.29
CD11b+	24,824	65.69
Macrophages	139	0.56
M1	56	40.29
M2	83	59.71
F4/80-	23,135	61.22
NK	1,319	5.70
NKT	13	0.06

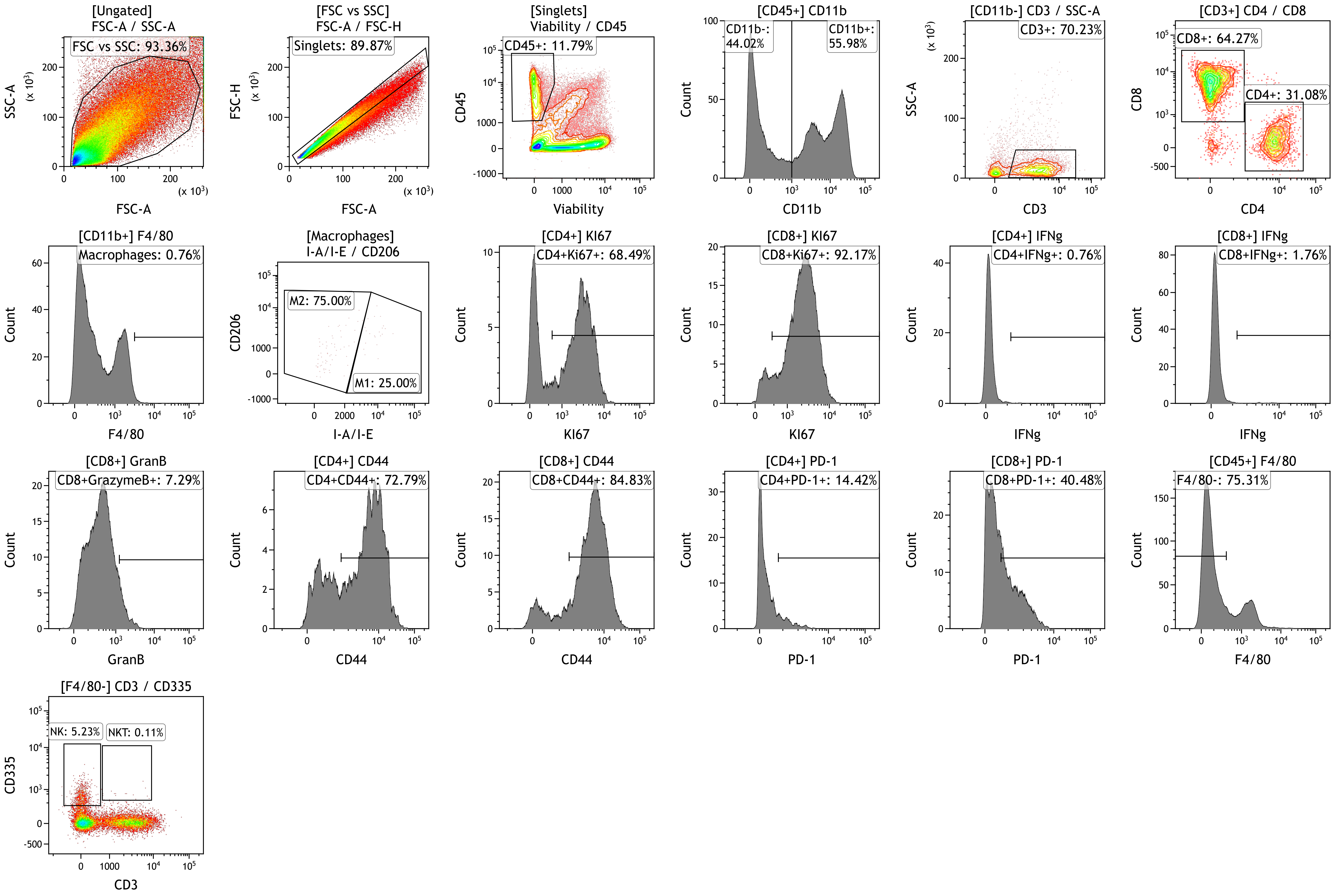

Gate	Number	%Gated
All	207,960	100.00
FSC vs SSC	194,157	93.36
Singlets	174,495	89.87
CD45+	20,573	11.79
CD11b-	9,057	44.02
CD3+	6,361	70.23
CD4+	1,977	31.08
CD4+CD44+	1,439	72.79
CD4+IFNg+	15	0.76
CD4+Ki67+	1,354	68.49
CD4+PD-1+	285	14.42
CD8+	4,088	64.27
CD8+CD44+	3,468	84.83
CD8+GrazymeB+	298	7.29
CD8+IFNg+	72	1.76
CD8+Ki67+	3,768	92.17
CD8+PD-1+	1,655	40.48
CD11b+	11,516	55.98
Macrophages	88	0.76
M1	22	25.00
M2	66	75.00
F4/80-	15,493	75.31
NK	811	5.23
NKT	17	0.11

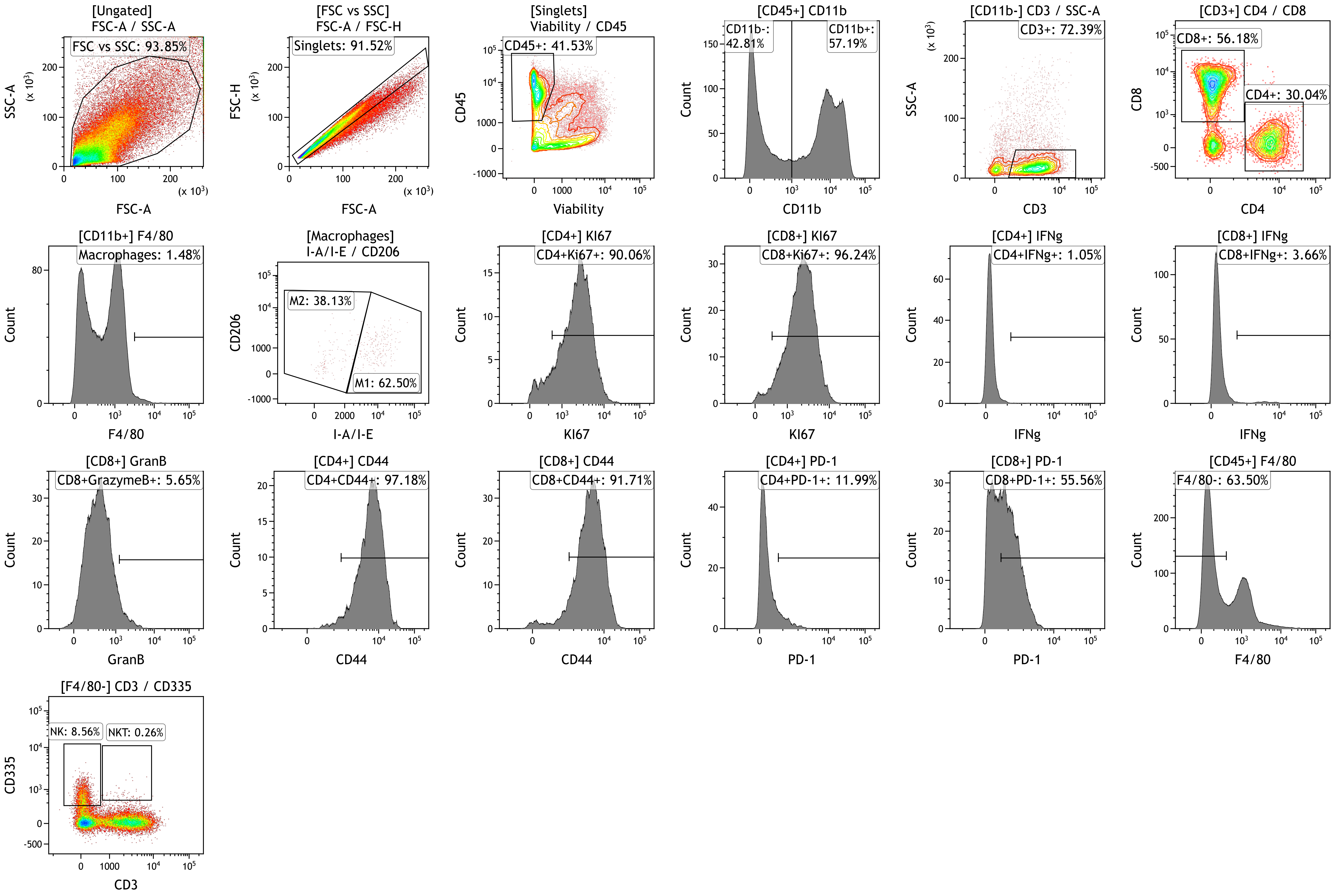

Gate	Number	%Gated
All	105,676	100.00
FSC vs SSC	99,175	93.85
Singlets	90,769	91.52
CD45+	37,698	41.53
CD11b-	16,140	42.81
CD3+	11,683	72.39
CD4+	3,510	30.04
CD4+CD44+	3,411	97.18
CD4+IFNg+	37	1.05
CD4+Ki67+	3,161	90.06
CD4+PD-1+	421	11.99
CD8+	6,564	56.18
CD8+CD44+	6,020	91.71
CD8+GrazymeB+	371	5.65
CD8+IFNg+	240	3.66
CD8+Ki67+	6,317	96.24
CD8+PD-1+	3,647	55.56
CD11b+	21,558	57.19
Macrophages	320	1.48
M1	200	62.50
M2	122	38.13
F4/80-	23,940	63.50
NK	2,049	8.56
NKT	62	0.26

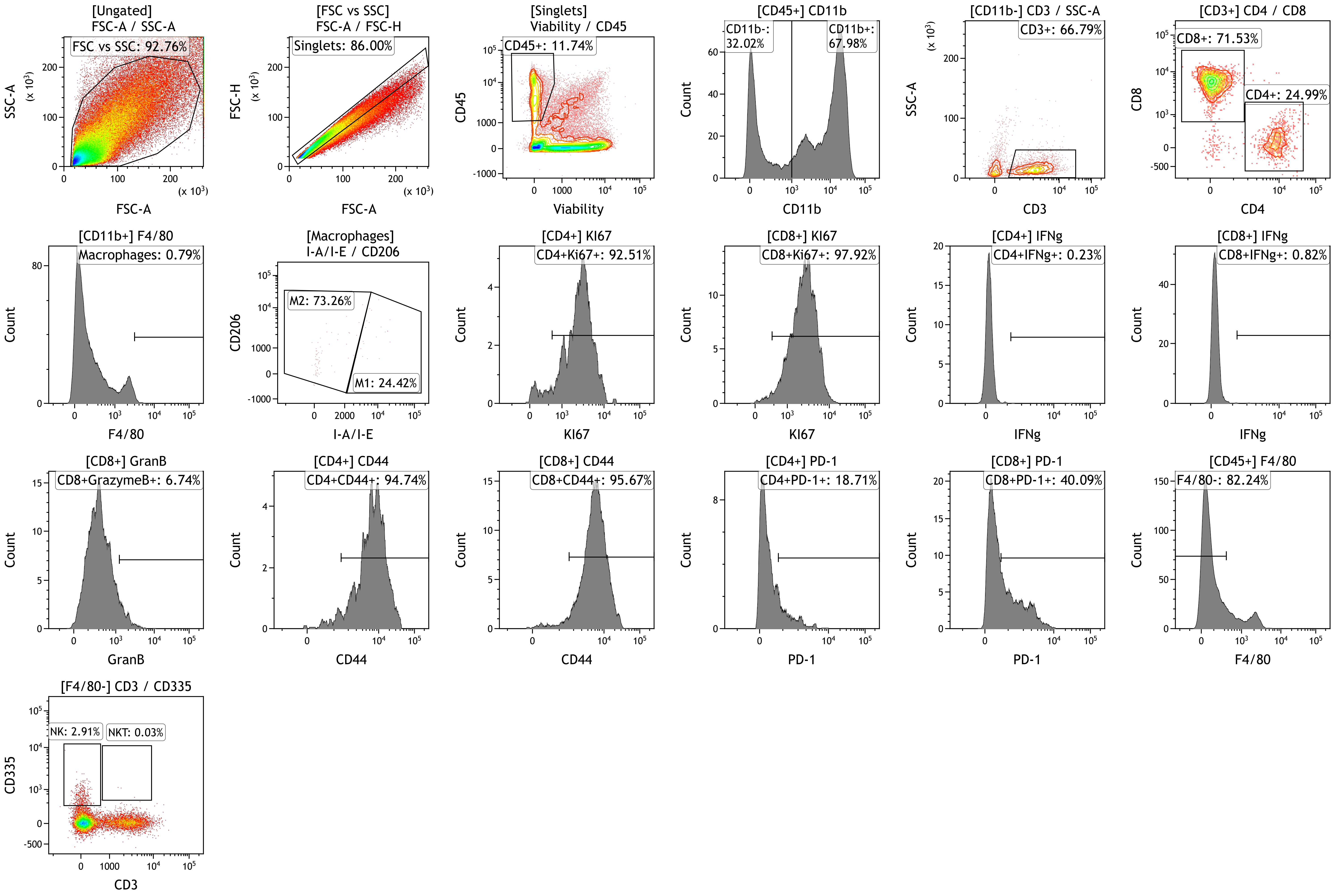

Gate	Number	%Gated
All	170,784	100.00
FSC vs SSC	158,425	92.76
Singlets	136,247	86.00
CD45+	15,997	11.74
CD11b-	5,122	32.02
CD3+	3,421	66.79
CD4+	855	24.99
CD4+CD44+	810	94.74
CD4+IFNg+	2	0.23
CD4+Ki67+	791	92.51
CD4+PD-1+	160	18.71
CD8+	2,447	71.53
CD8+CD44+	2,341	95.67
CD8+GrazymeB+	165	6.74
CD8+IFNg+	20	0.82
CD8+Ki67+	2,396	97.92
CD8+PD-1+	981	40.09
CD11b+	10,875	67.98
Macrophages	86	0.79
M1	21	24.42
M2	63	73.26
F4/80-	13,156	82.24
NK	383	2.91
NKT	4	0.03

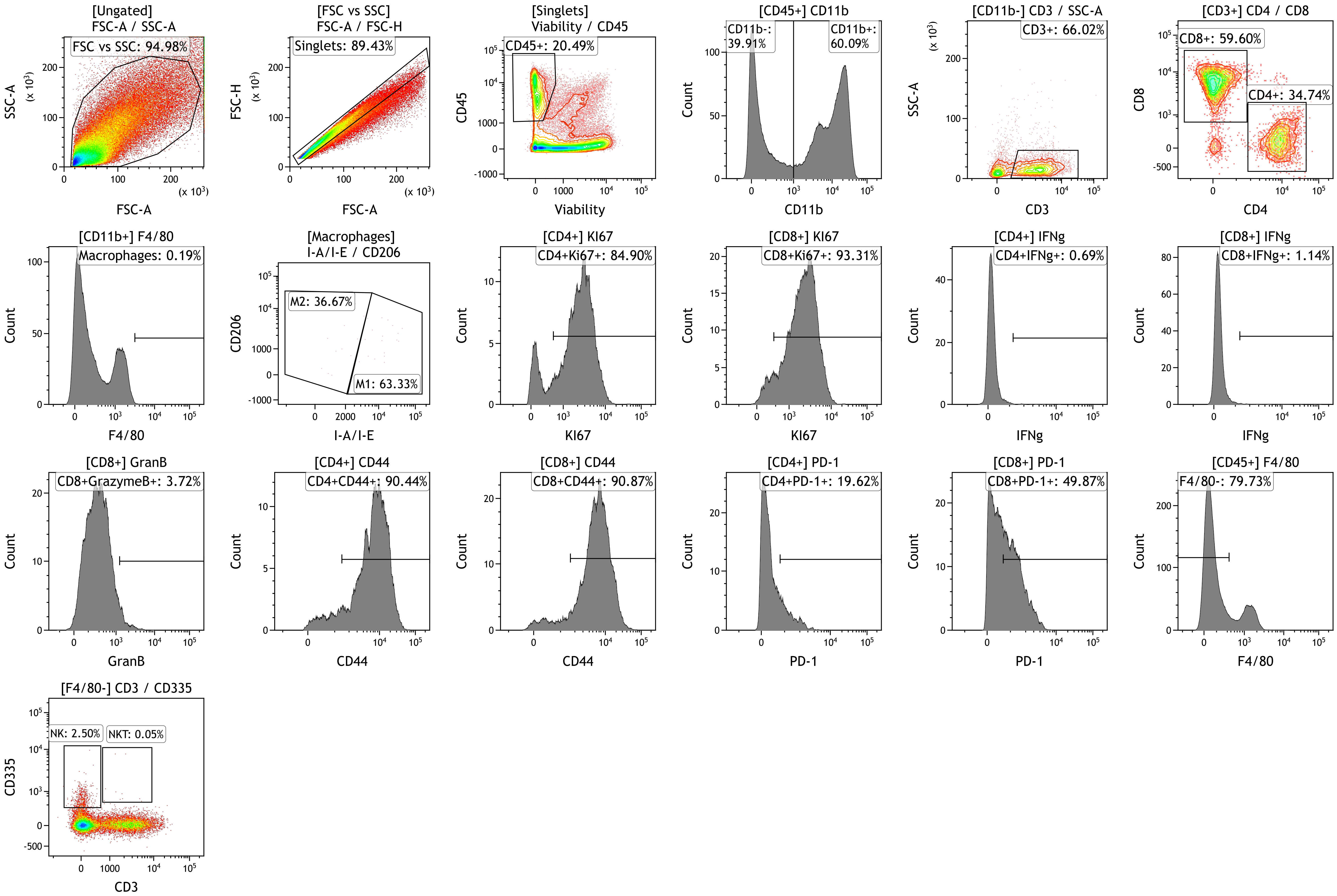

Gate	Number	%Gated
All	154,176	100.00
FSC vs SSC	146,434	94.98
Singlets	130,962	89.43
CD45+	26,839	20.49
CD11b-	10,712	39.91
CD3+	7,072	66.02
CD4+	2,457	34.74
CD4+CD44+	2,222	90.44
CD4+IFNg+	17	0.69
CD4+Ki67+	2,086	84.90
CD4+PD-1+	482	19.62
CD8+	4,215	59.60
CD8+CD44+	3,830	90.87
CD8+GrazymeB+	157	3.72
CD8+IFNg+	48	1.14
CD8+Ki67+	3,933	93.31
CD8+PD-1+	2,102	49.87
CD11b+	16,127	60.09
Macrophages	30	0.19
M1	19	63.33
M2	11	36.67
F4/80-	21,400	79.73
NK	536	2.50
NKT	10	0.05

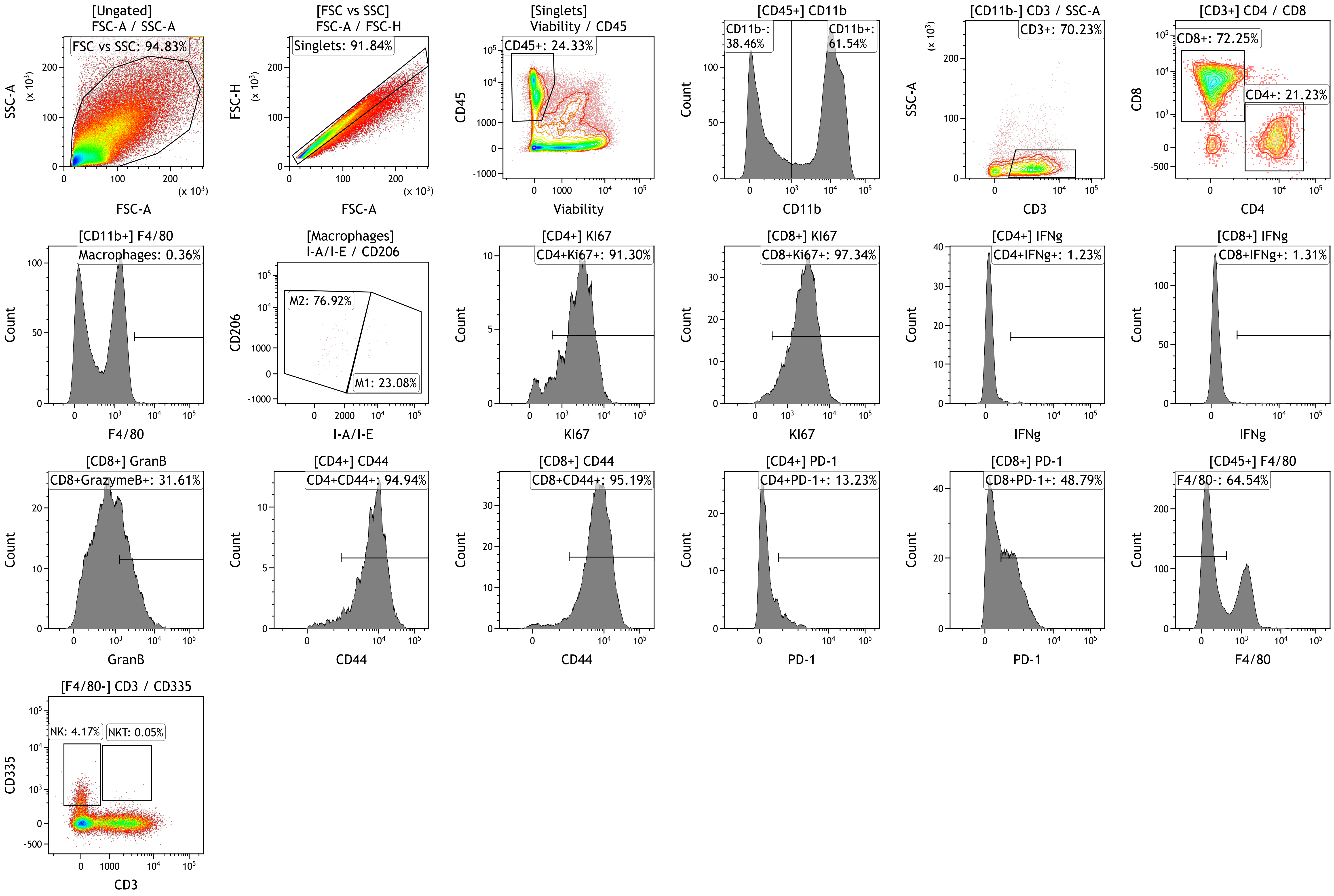

Gate	Number	%Gated
All	167,376	100.00
FSC vs SSC	158,728	94.83
Singlets	145,779	91.84
CD45+	35,469	24.33
CD11b-	13,640	38.46
CD3+	9,580	70.23
CD4+	2,034	21.23
CD4+CD44+	1,931	94.94
CD4+IFNg+	25	1.23
CD4+Ki67+	1,857	91.30
CD4+PD-1+	269	13.23
CD8+	6,922	72.25
CD8+CD44+	6,589	95.19
CD8+GrazymeB+	2,188	31.61
CD8+IFNg+	91	1.31
CD8+Ki67+	6,738	97.34
CD8+PD-1+	3,377	48.79
CD11b+	21,829	61.54
Macrophages	78	0.36
M1	18	23.08
M2	60	76.92
F4/80-	22,892	64.54
NK	954	4.17
NKT	11	0.05

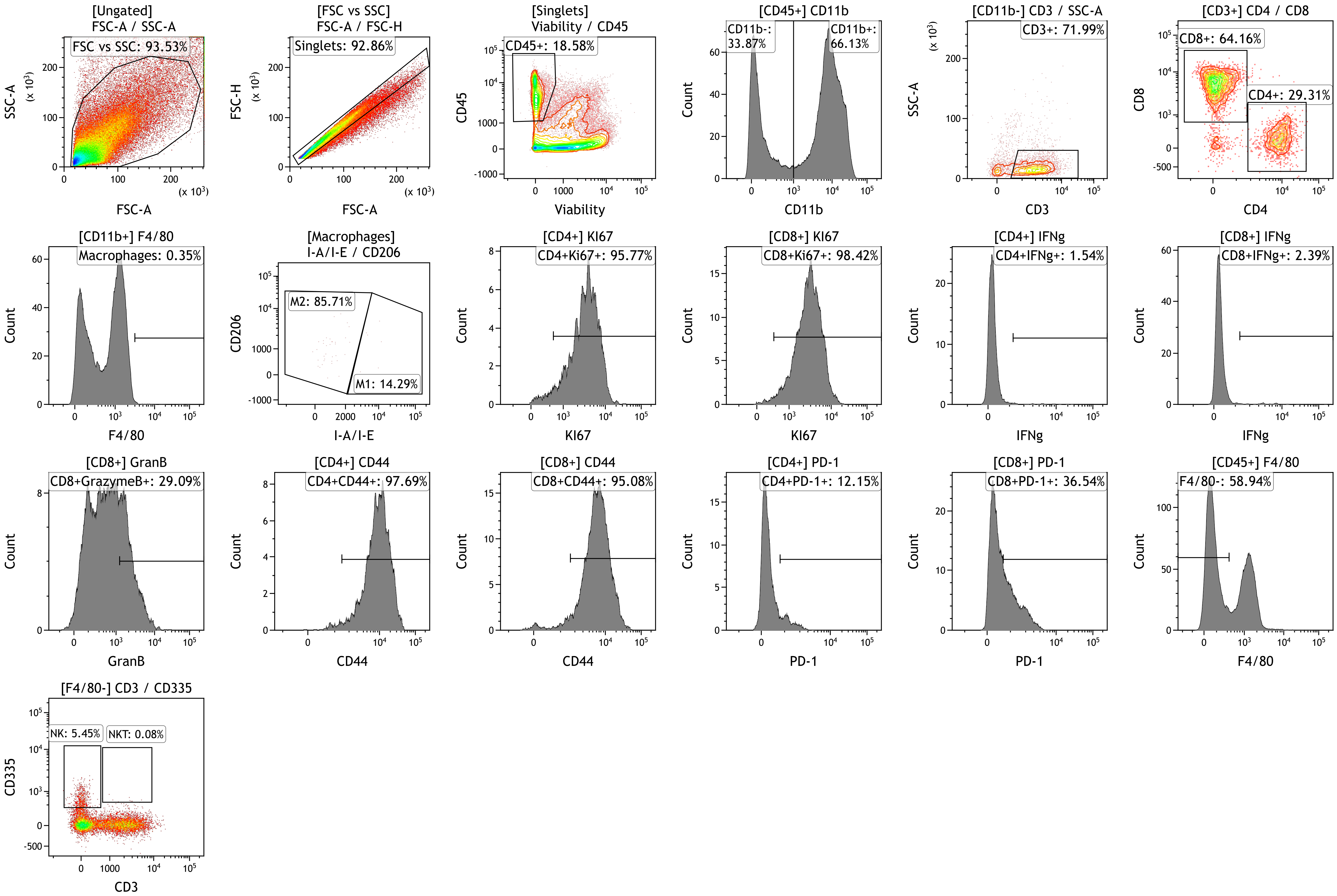

Gate	Number	%Gated
All	112,728	100.00
FSC vs SSC	105,429	93.53
Singlets	97,903	92.86
CD45+	18,191	18.58
CD11b-	6,162	33.87
CD3+	4,436	71.99
CD4+	1,300	29.31
CD4+CD44+	1,270	97.69
CD4+IFNg+	20	1.54
CD4+Ki67+	1,245	95.77
CD4+PD-1+	158	12.15
CD8+	2,846	64.16
CD8+CD44+	2,706	95.08
CD8+GrazymeB+	828	29.09
CD8+IFNg+	68	2.39
CD8+Ki67+	2,801	98.42
CD8+PD-1+	1,040	36.54
CD11b+	12,029	66.13
Macrophages	42	0.35
M1	6	14.29
M2	36	85.71
F4/80-	10,722	58.94
NK	584	5.45
NKT	9	0.08

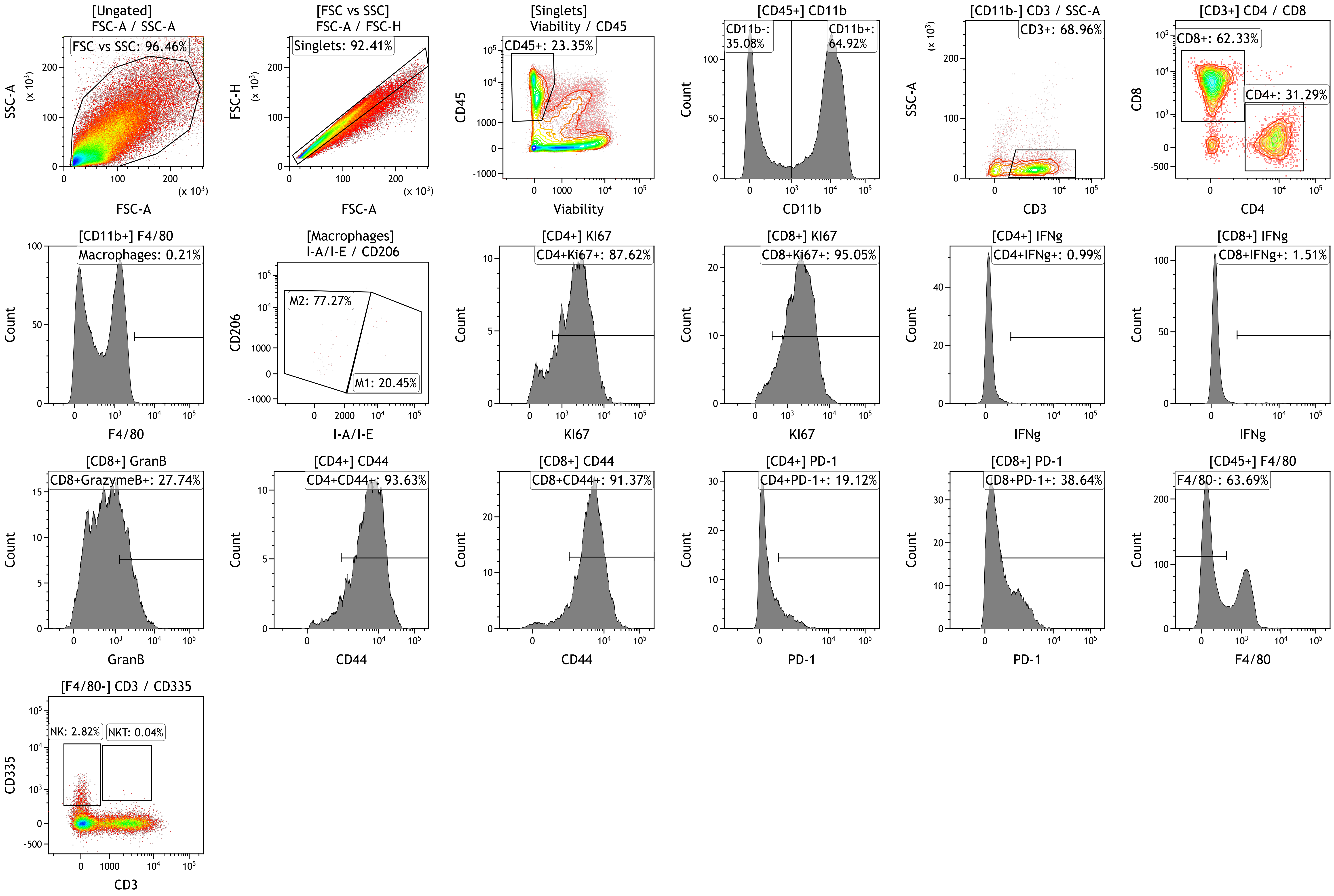

Gate	Number	%Gated
All	154,344	100.00
FSC vs SSC	148,886	96.46
Singlets	137,591	92.41
CD45+	32,128	23.35
CD11b-	11,272	35.08
CD3+	7,773	68.96
CD4+	2,432	31.29
CD4+CD44+	2,277	93.63
CD4+IFNg+	24	0.99
CD4+Ki67+	2,131	87.62
CD4+PD-1+	465	19.12
CD8+	4,845	62.33
CD8+CD44+	4,427	91.37
CD8+GrazymeB+	1,344	27.74
CD8+IFNg+	73	1.51
CD8+Ki67+	4,605	95.05
CD8+PD-1+	1,872	38.64
CD11b+	20,856	64.92
Macrophages	44	0.21
M1	9	20.45
M2	34	77.27
F4/80-	20,463	63.69
NK	578	2.82
NKT	9	0.04
